# Supplementary material for: Characterizing and controlling CRISPR repair outcomes in nondividing human cells
Source: Nat Commun. 2025 Nov 17;16:9883. doi: 10.1038/s41467-025-66058-3 (PMC12623481; doi:10.1038/s41467-025-66058-3)
Supplement: Supplementary file 1 — Supplementary Information [file 41467_2025_66058_MOESM1_ESM.pdf]

SUPPLEMENTARY FIGURES

Characterizing and controlling CRISPR repair outcomes in nondividing human cells

Gokul N Ramadoss[1,2], Samali J Namaganda[1], Manasi Kumar[1], Jennifer R Hamilton[3,4], Rohit Sharma[3,5], Karena G Chow[1], Luke A Workley[1,6], Bria L Macklin[1], Mengyuan Sun[1], Alvin S Ha[1,6], Jia-Cheng Liu[7], Christof Fellmann[1,8], Hannah L Watry[1], Philip H Dierks[1], Rudra S Bose[2], Julianne Jin[2], Barbara S Perez[3,4], Cindy R Sandoval Espinoza[3,4], Madeline P Matia[1], Serena H Lu[1], Luke M Judge[1,9], Brian R Shy[1,6,10], Andre Nussenzweig[7], Britt Adamson[11,12], Niren Murthy[3,5], Jennifer A Doudna[1,3,4,13,14,15,16], Martin Kampmann[2,17], Bruce R Conklin[1,3,7,18,†]

1. Gladstone Institutes, San Francisco, CA, 94158, USA.
2. Institute for Neurodegenerative Diseases, University of California, San Francisco, CA, 94158, USA.
3. Innovative Genomics Institute, University of California, Berkeley, CA, 94720, USA.
4. Department of Molecular & Cell Biology, University of California, Berkeley, CA, 94720, USA.
5. Department of Bioengineering, University of California, Berkeley, CA, 94720, USA.
6. Department of Laboratory Medicine, University of California, San Francisco, CA, 94158, USA
7. Laboratory of Genome Integrity, National Cancer Institute, NIH, Bethesda, MD, 20892, USA.
8. Department of Cellular & Molecular Pharmacology, University of California, San Francisco, CA, 94158, USA.
9. Department of Pediatrics, University of California, San Francisco, CA, 94158, USA.
10. Helen Diller Family Comprehensive Cancer Center, University of California, San Francisco, 94158, CA, USA
11. Department of Molecular Biology, Princeton University, Princeton, NJ, 08544, USA.
12. Lewis–Sigler Institute for Integrative Genomics, Princeton University, Princeton, NJ, 08544, USA.
13. California Institute for Quantitative Biosciences, University of California, Berkeley, CA, 94720, USA.
14. Howard Hughes Medical Institute, University of California, Berkeley, CA, 94720, USA.
15. Department of Chemistry, University of California, Berkeley, CA, 94720, USA.
16. MBIB Division, Lawrence Berkeley National Laboratory, Berkeley, CA, 94720, USA.
17. Department of Biochemistry & Biophysics, University of California, San Francisco, CA, 94158, USA.
18. Department of Medicine, University of California, San Francisco, CA, 94158, USA.

† Corresponding author: Bruce R Conklin, bconklin@gladstone.ucsf.edu

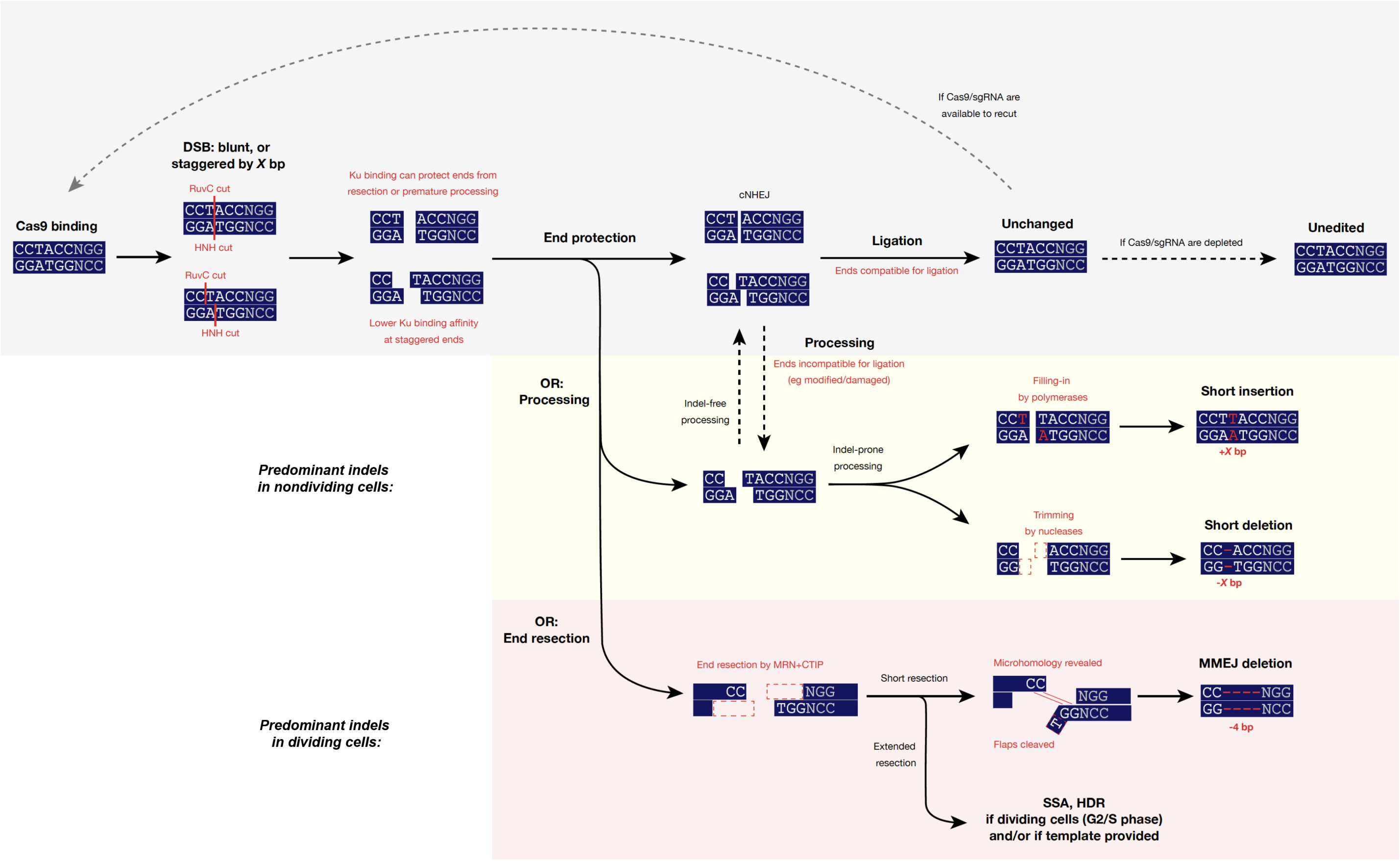

Supplementary Figure 1: Schematic of how DSB repair pathways determine the CRISPR editing outcome. Cas9 induces a blunt or staggered DSB, depending on where the RuvC domain cleaves (Shou et al, Mol Cell, 2018. PMID: 30033371). The exposed DNA ends are then subjected to either end protection or end resection (or other processing). End protection generally leads to cNHEJ. If the protected ends are still chemically compatible for ligation, cNHEJ often ligates them fidelitously, yielding an unchanged sequence which can be re-cut by any remaining Cas9 RNP. If the protected ends are not compatible for ligation, or if end protection was outcompeted by processing machinery such as polymerases and nucleases, then NHEJ processing can occur (Stinson et al, Mol Cell, 2020. PMID: 31862156). This processing sometimes introduces indels. In dividing cells, end resection often outcompetes end protection, leading to resection-dependent pathways such as MMEJ, HDR and SSA. Resection-dependent pathways can cause indels (MMEJ/SSA) or templated repair (HDR).

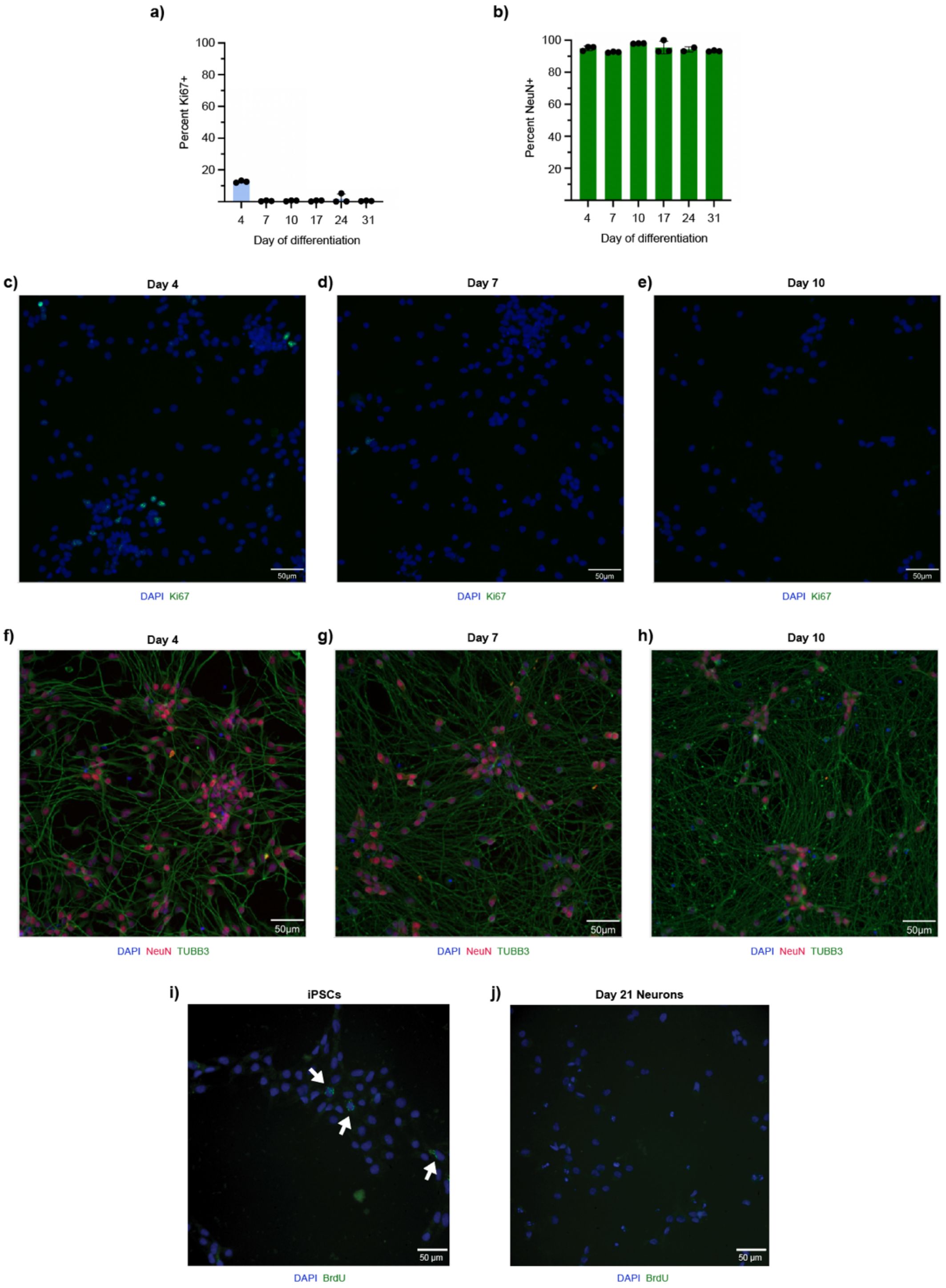

Supplementary Figure 2: Characterizing the purity of the neuronal differentiation.
a) By Day 7 of differentiation, less than 1% of cells are proliferative (Ki67+). Bars show what percentage of DAPI+ nuclei were Ki67+, averaged across 3 replicate wells. Quantified using HCS Studio SpotDetector. **b)** By Day 4 of differentiation, 95% of cells express a neuron-specific marker (NeuN+). Bars show what percentage of DAPI+ nuclei were NeuN+, averaged across 3 replicate wells. Quantified using CellProfiler. For a-b: Each dot is one replicate well, totaled across 13 non-overlapping fields per well. Error bars show SEM. **c-e)** Representative ICC images showing DAPI and Ki67 staining from Days 4/7/10 of differentiation; quantified in a. **f-h)** Representative ICC images showing DAPI, NeuN, and TUBB3 staining from Days 4/7/10 of differentiation; quantified in b. TUBB3 is another marker of mature neurons. **i-j)** Representative ICC images showing BrdU incorporation in iPSCs but not neurons. Cells were treated with 10μM BrdU for 2 h at 37C, then fixed and stained with mouse BrdU mAb (CST #5292), followed by secondary antibody (Invitrogen, #A11001) and DAPI. **Note for c-j**: Scale bars were added manually based on the images' pixel:micron ratio.

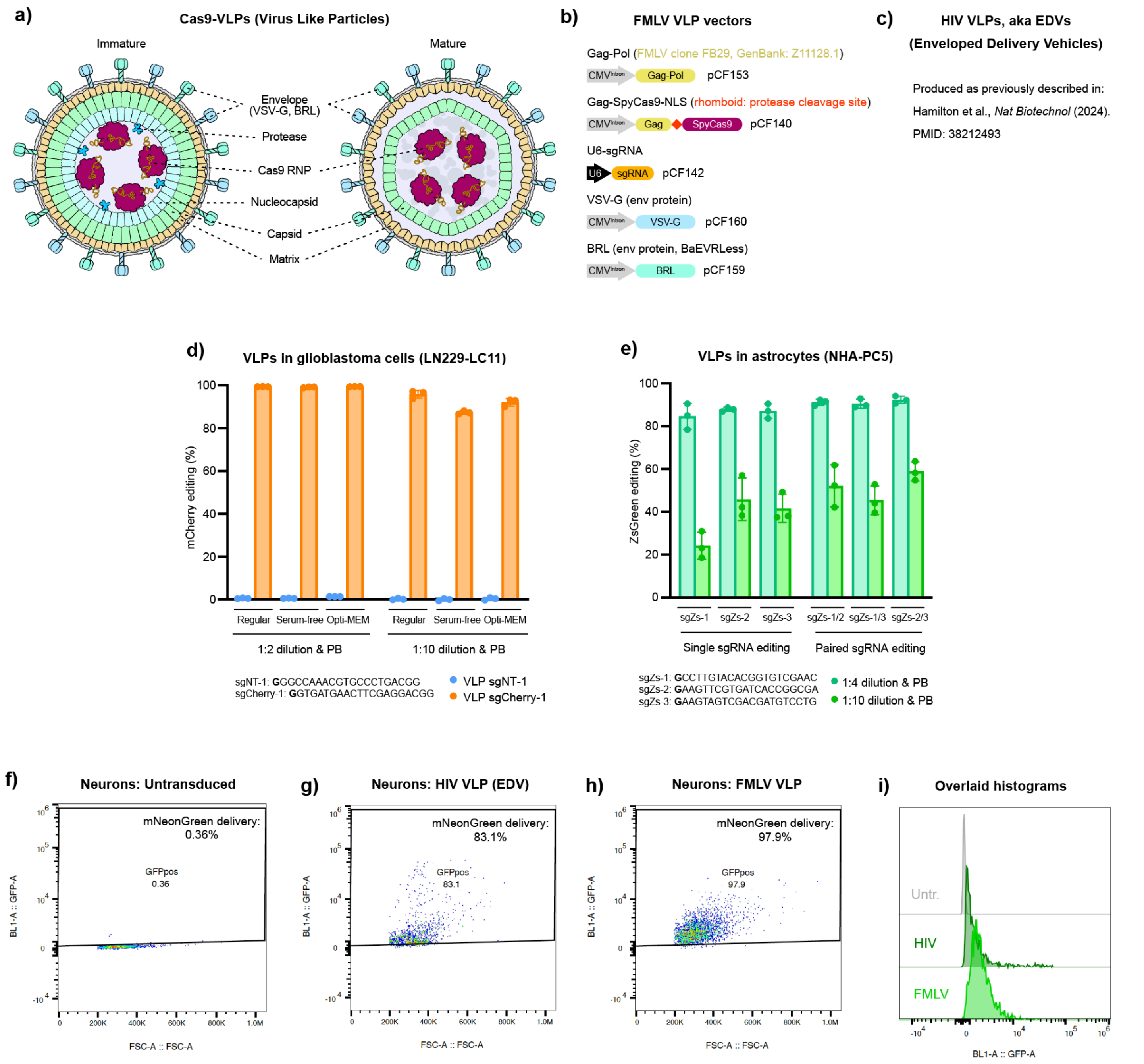

Supplementary Figure 3: Establishing VLP delivery of Cas9 to human postmitotic neurons.
a) Schematic depicting the components of virus-like particles. Matrix, capsid, and nucleocapsid are part of the Gag polyprotein. VSV-G and BRL are envelope (env) proteins for pseudotyping to mediate broad and efficient cellular transduction. **b)** Maps and nomenclature of optimized FMLV VLP vectors. **c)** Vectors used to produce HIV VLPs, also known as enveloped delivery vehicles (EDVs), were previously described in Hamilton et al., Nat Biotechnol, 2024. PMID: 38212493. **d)** Assessment of editing efficiency with optimized FMLV VLPs in glioblastoma cells. Monoclonal mCherry-expressing glioblastoma cells (LN229-LC11) were transduced with the indicated VLPs harvested in regular growth media, serum-free growth media, or Opti-MEM. Target cells were transduced at the indicated VLP dilution, with addition of polybrene (PB, 5 µg/ml). At day six post-transduction, mCherry editing efficiency (mCherry-) was assessed by flow cytometry. Non-transduced cells were used for normalization. VLP sgCherry-1: CRISPR-Cas9 VLP containing a previously validated mCherry-targeting sgRNA (Knott et al, eLife, 2019. PMID: 31397669). VLP sgNT-1: CRISPR-Cas9 VLP containing a non-targeting control sgRNA. Error bars indicate standard deviation. **e)** Assessment of editing efficiency with optimized FMLV VLPs in astrocytes. Normal human astrocytes expressing ZsGreen (NHA-PC5), and previously

treated with puromycin-targeting VLPs (Tan et al, Cell Reports, 2023. PMID: 37917583), were transduced with sgZsGreen-targeting VLPs (harvested in regular growth media) at the indicated dilution, with addition of polybrene (PB). Cells were either transduced with a single VLP to generate indels or with a mixture of two VLPs to induce a deletion in ZsGreen. At day six post-transduction, ZsGreen editing efficiency (ZsGreen-) was assessed by flow cytometry. Non-transduced cells were used for normalization. VLP sgZs-1/2/3: CRISPR-Cas9 VLPs containing ZsGreen-targeting sgRNAs. Error bars indicate standard deviation. **f-i)** Our optimized FMLV VLPs, and HIV VLPs (EDVs), both transduced human iPSC-derived neurons efficiently. Flow cytometry 1-week post-transduction with no VLP (untransduced), HIV VLPs, or optimized FMLV VLPs shows up to 97% maximum transduction efficiency. Dose: 2 μL VLP per 100 μL media.

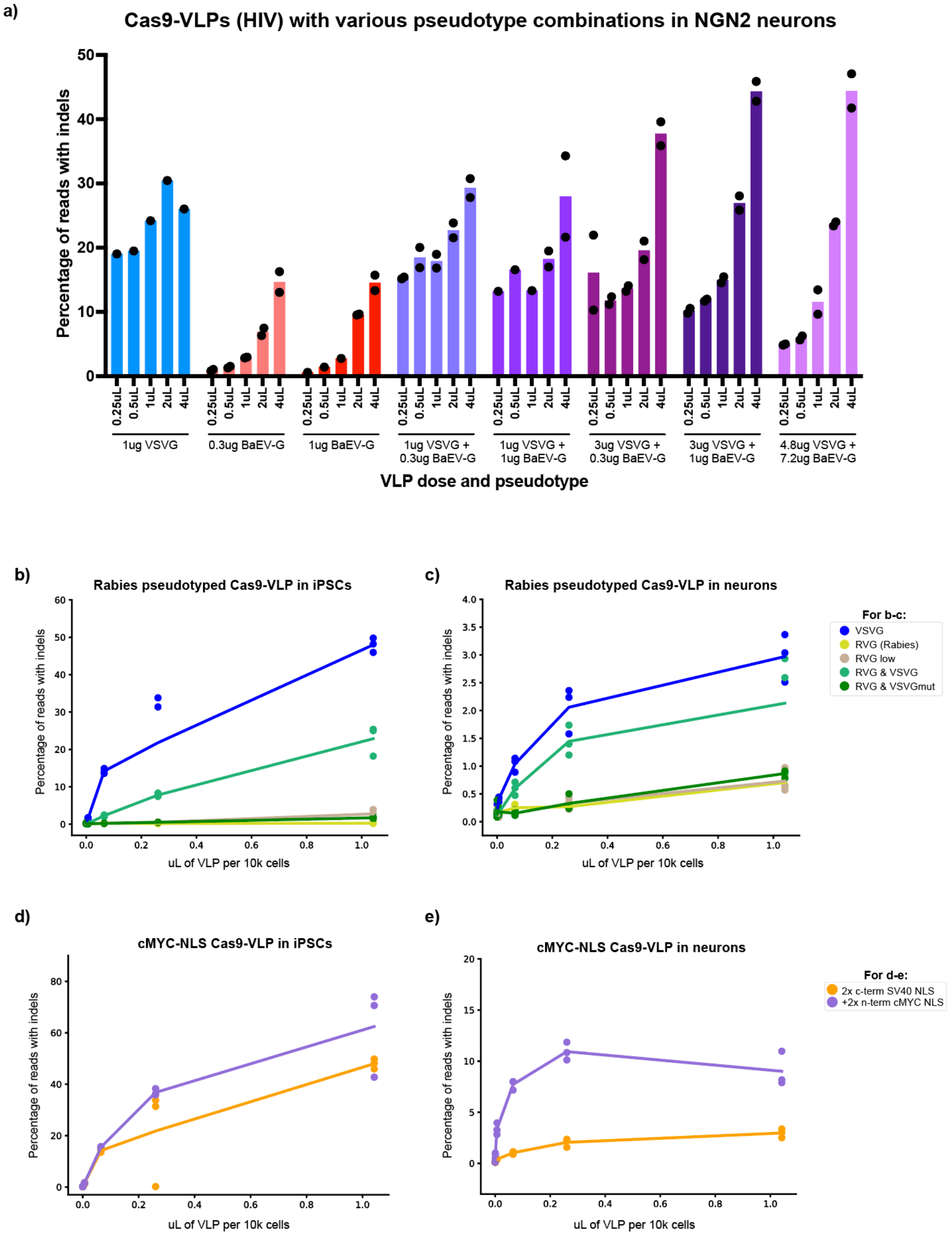

Supplementary Figure 4: Altering the pseudotype and NLS of Cas9-VLPs can significantly improve editing efficiency. a) Neuron editing efficiency (B2Mg1) of HIV Cas9-VLPs pseudotyped with various combinations of viral glycoproteins, across a serial dilution of VLP doses per 100 µL media. VSVG alone appears more effective at lower VLP doses, while co-pseudotyping with BaEV-G appears more effective at higher VLP doses. This may suggest that VSVG is more potent but its surface receptor (LDLR) on neurons gets saturated earlier than that of BAEV-G. In subsequent experiments with HIV VLPs, we kept the original 1 µg VSVG pseudotype for HIV VLPs (per Hamilton et al, Cell Rep, 2021, PMCID: PMC8236216). In subsequent experiments with FMLV VLPs, we used a 4.8 µg / 7.2 µg ratio of VSVG to BaEV-G R-less (BRL), similar to the rightmost condition shown in a. **b-c)** Pseudotyping/co-pseudotyping HIV VLPs with Rabies Virus G protein (RVG) did not improve editing efficiency in neurons, nor in iPSCs. **d-e)** Adding 2x cMYC NLS tags on the N-terminus of Cas9 significantly improved editing efficiency of HIV Cas9-VLPs in both iPSCs and neurons, compared to Cas9 with only the standard 2x C-terminus SV40 NLS. For a: CRISPResso2 analysis of amplicon-NGS, 4 days post-transduction. For b-e: Synthego ICEv2 analysis, 3 days post-transduction.

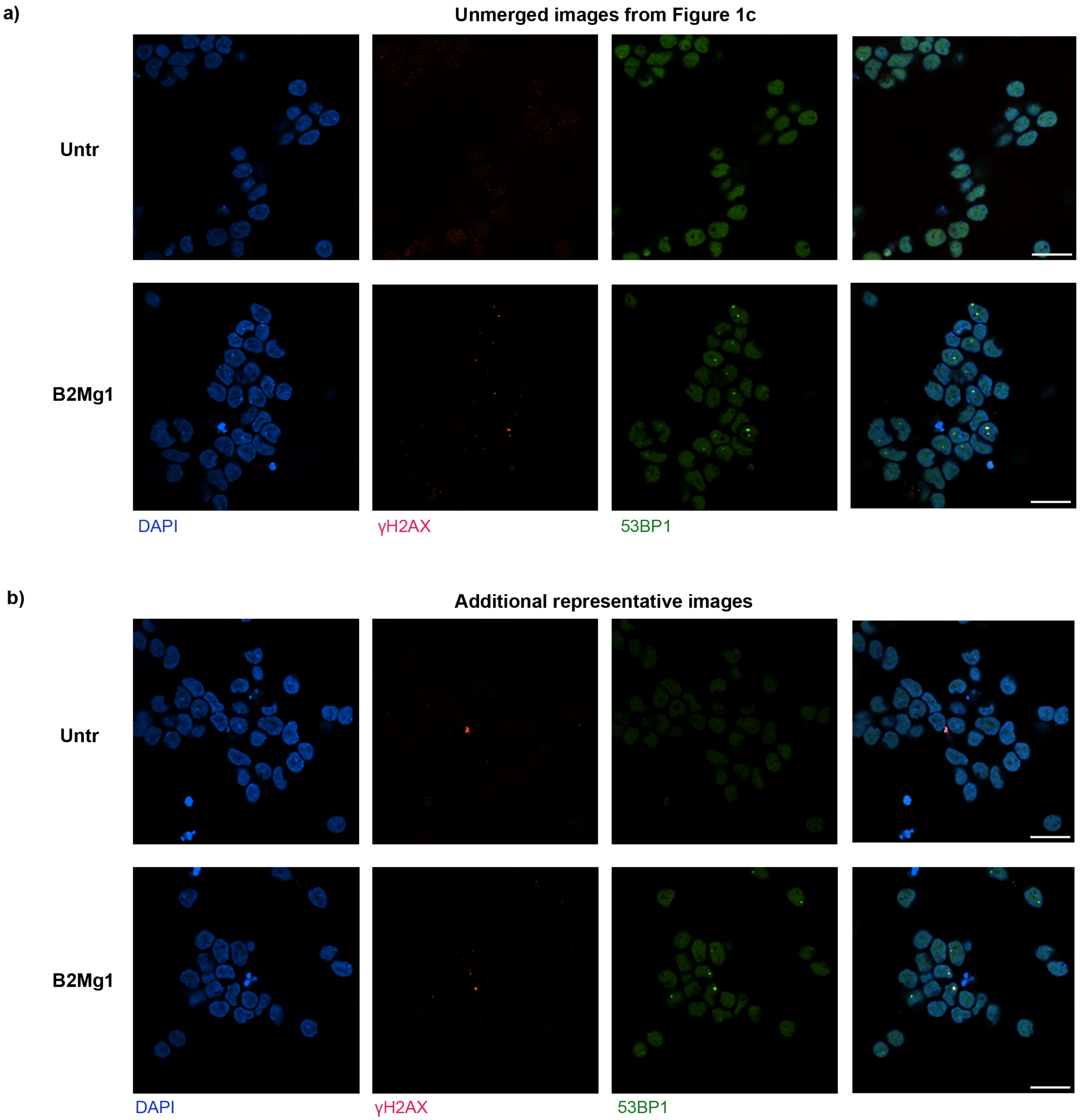

Supplementary Figure 5: Cas9-VLPs induce DSBs in human postmitotic neurons.
a) Unmerged panels from Figure 1c, showing DSBs induced by Cas9-VLPs in human iPSC-derived neurons, compared to age-matched untransduced neurons. For a-b: Neurons transduced 2 weeks into differentiation, and imaged 3 days post-transduction. DSBs are co-labeled by markers γH2AX (red) and 53BP1 (green). Dose: 1 μL FMLV VLP per 100 μL media. Scale bar is 20 μm. **b)** Additional representative ICC images showing DSBs induced by Cas9-VLPs in human iPSC-derived neurons, compared to age-matched untransduced neurons.

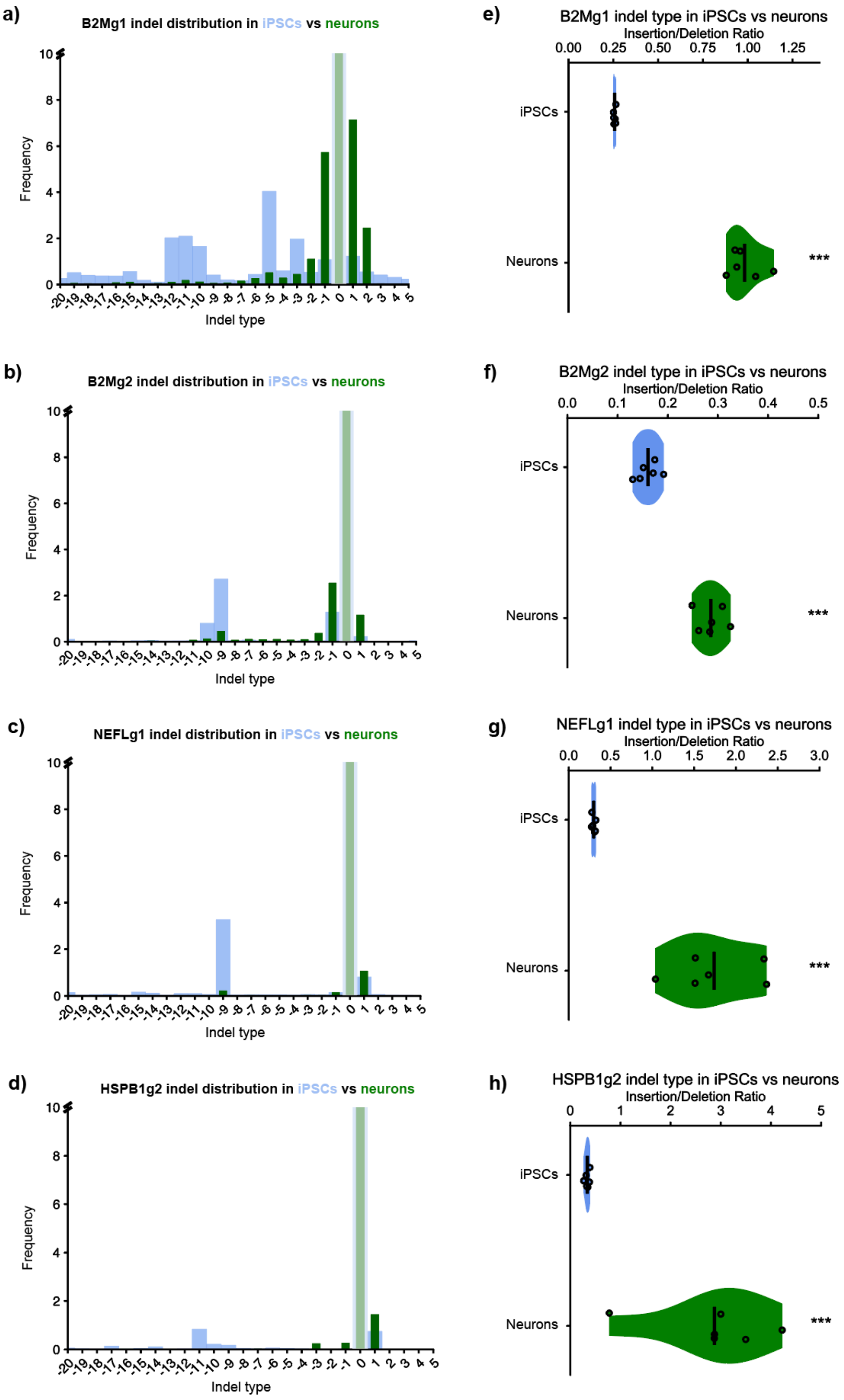

Supplementary Figure 6: CRISPR editing outcomes differ in nondividing neurons compared to dividing iPSCs. a-d) For each of four separate sgRNAs, CRISPR editing outcomes differ between nondividing neurons and dividing iPSCs (genetically identical). Despite differences in which indel outcomes each sgRNA was amenable to overall, in each case, the MMEJ-like deletions were predominant in iPSCs whereas the NHEJ-like small indels were predominant in neurons. Dose: 2 µL VLP (FMLV) per 100 µL media. Average of 6 replicate wells, transduced in parallel. Genomic DNA was harvested 5 days post-transduction, processed for amplicon-NGS, then analyzed by CRISPResso2. **e-h)** Quantifying the differences shown in a-d respectively, by calculating the ratio of total insertions to total deletions in each condition. Neurons compared to iPSCs, One Factor ANOVA, *** $p<0.0005$.

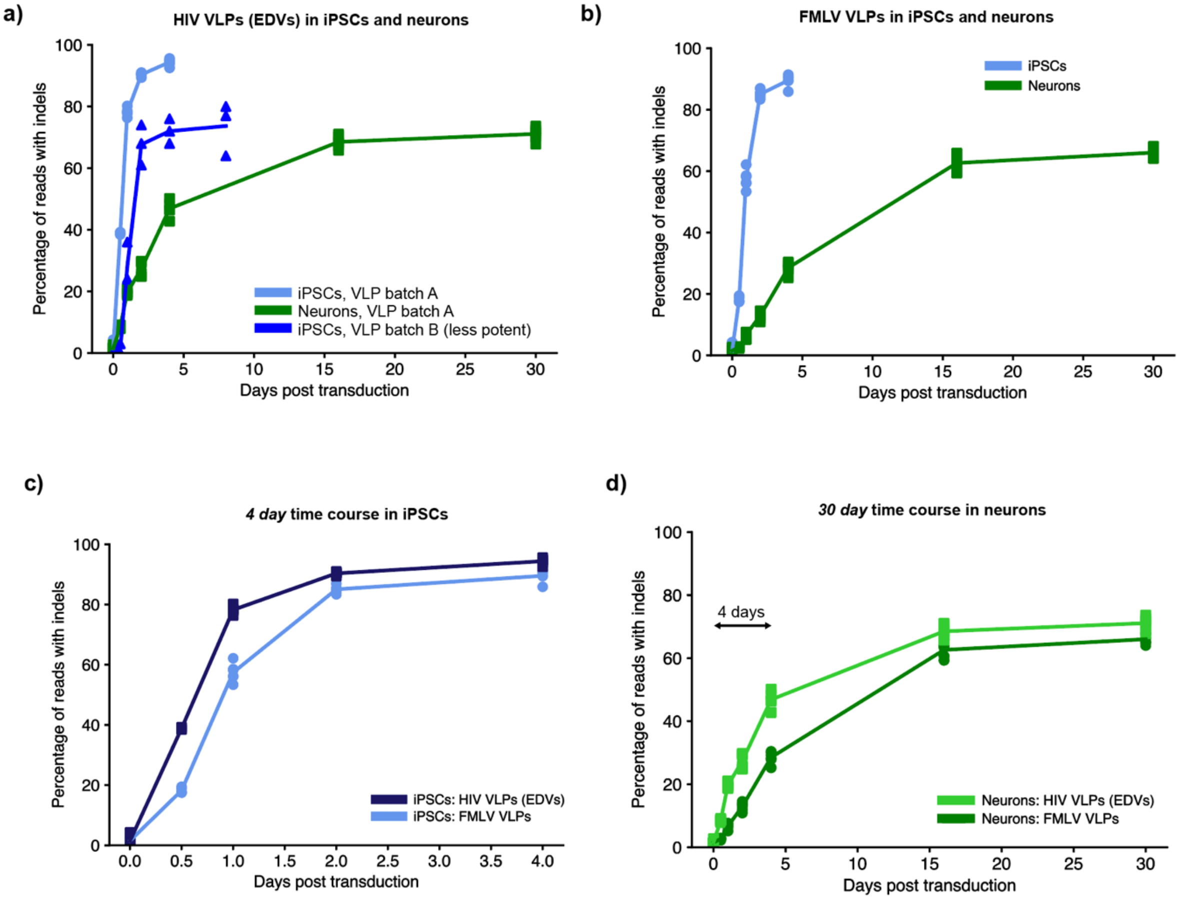

Supplementary Figure 7: Cas9-VLP-induced indels accumulate for weeks post-transduction in neurons.
a) Even in experiments where iPSCs plateaued at a lower editing efficiency, they still reached that plateau sooner than neurons. Regraphed data from Figure 2a, and overlayed data from a separate experiment with a less-efficient batch of VLPs. In this experiment, iPSCs plateaued at 60-70% indels instead of 90%+, but still reached that plateau within ~4 days. **b)** The overlaid time courses are reproduced very comparably with FMLV VLPs compared to HIV VLPs (a, and Figure 2a). **c-d)** With either HIV or FMLV Cas9-VLPs, indels plateaued within 4 days post-transduction in iPSCs (c), but continued to increase for up to 16 days post-transduction in neurons (d). Dose: 2 µL VLP per 100 µL media. CRISPResso2 analysis of amplicon-NGS.

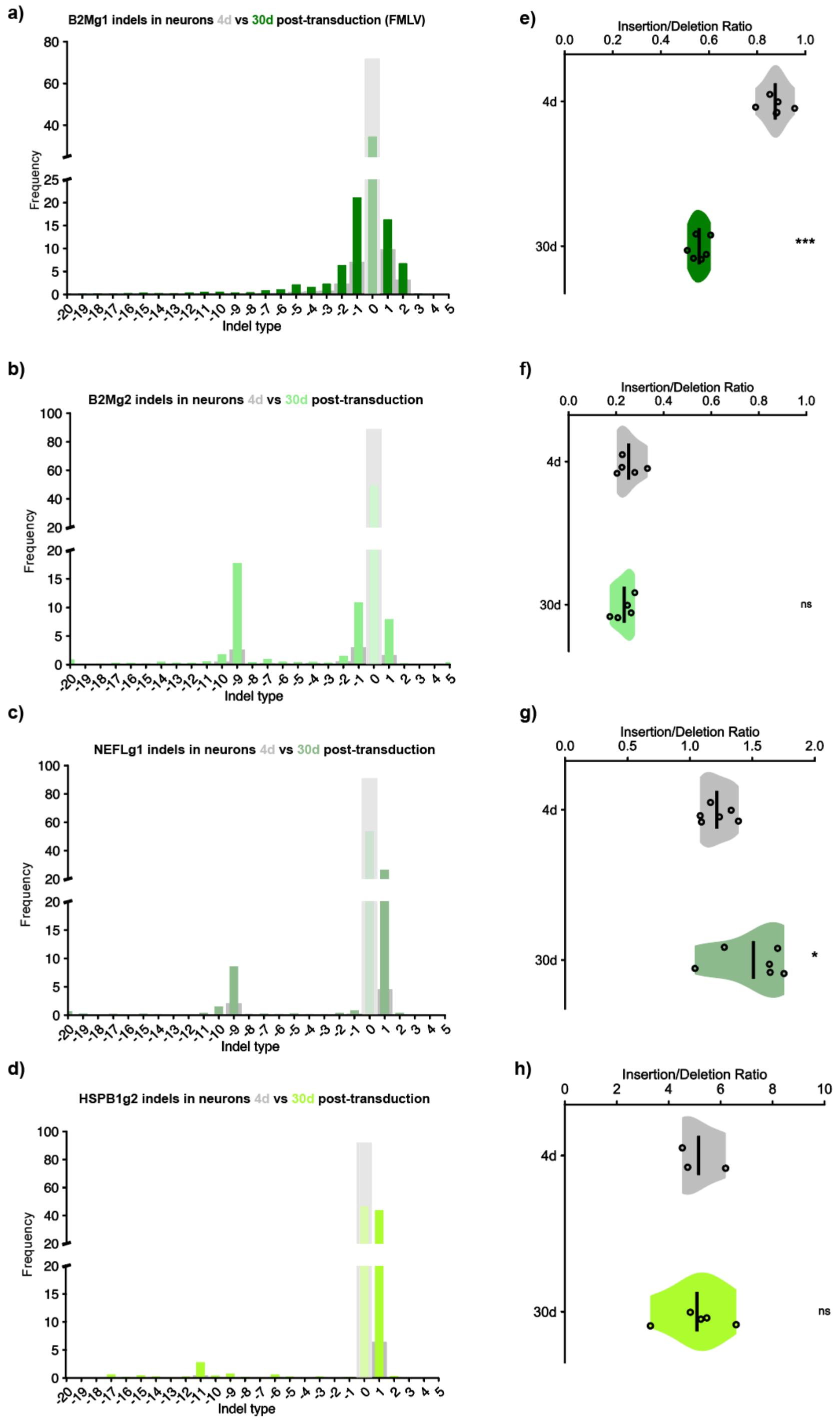

Supplementary Figure 8: Distribution of indel types can sometimes change between early and late timepoints in Cas9-VLP-treated neurons.

a-d) Across four different sgRNAs, despite differences in which indel outcomes each sgRNA was amenable to overall, all available indel outcomes at the 4 day timepoint increased in prevalence by the 30 day timepoint. Dose: 1 µL VLP (FMLV) per 100 µL media. **e-h)** Depending on the sgRNA, the ratio of insertions to deletions *can* change between 4 days and 30 days post-transduction in neurons – but there is no *generalizable* trend of which indel types are more prevalent early vs late. Quantification of total insertions and total deletions visualized in a-d respectively. 30 d timepoint compared to 4 d timepoint, One Factor ANOVA, * p<0.05, *** p<0.0005, ns = not significant. CRISPResso2 analysis of amplicon-NGS.

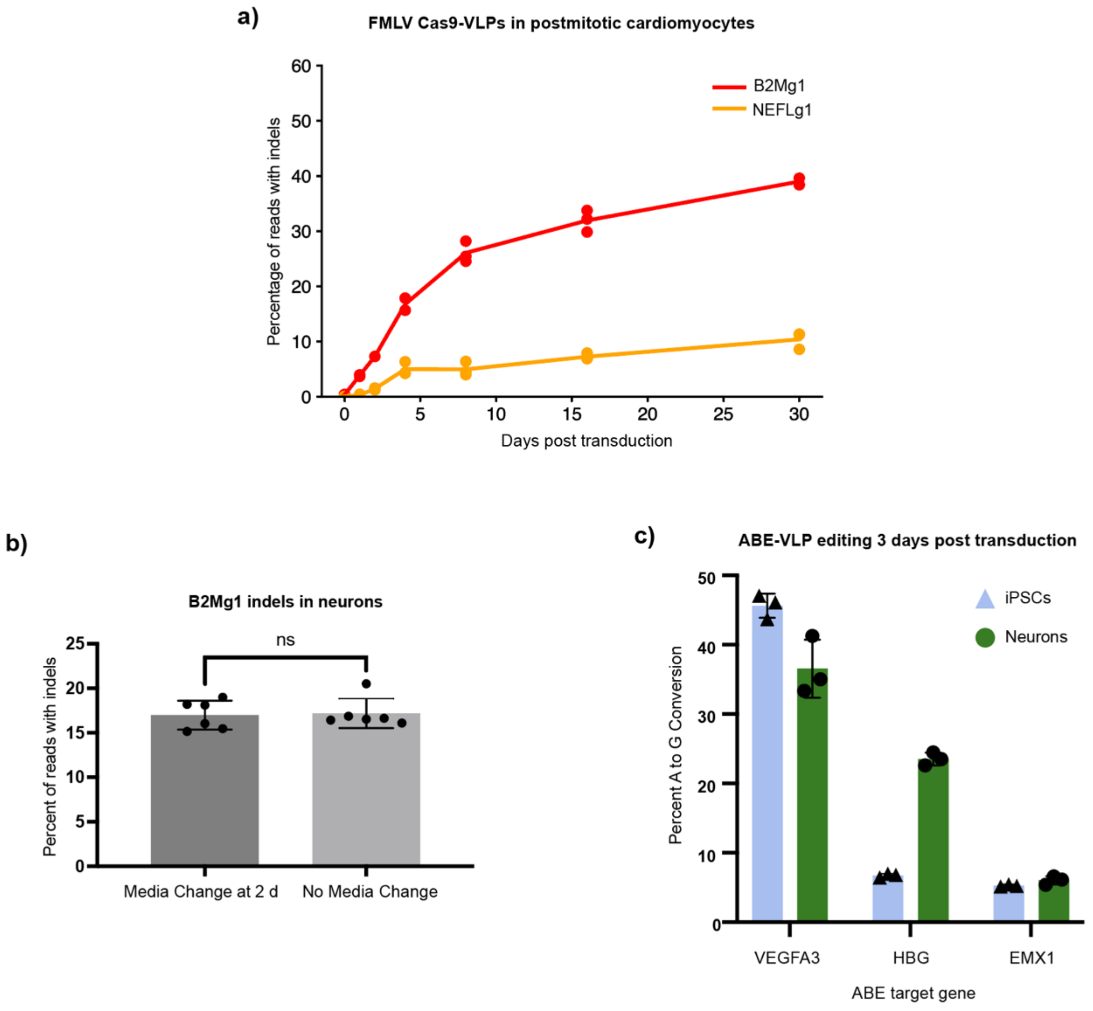

Supplementary Figure 9: Testing additional hypotheses about the prolonged editing time course in neurons.
a) Postmitotic iPSC-derived cardiomyocytes (CMs) also show a weeks-long accumulation of indels, for two different sgRNAs. At day 30+ of differentiation, after lactate purification to select for postmitotic CMs, CMs were transduced with 1 μL FMLV VLP per 100 μL media. 3 replicate wells per condition, transduced in parallel. CRISPResso2 analysis of amplicon-NGS. CMs were generated from WTC background iPSCs using the protocol described in Perez-Bermejo et al, Sci Transl Med, 2021 (PMCID: PMC8128284). **b)** The prolonged indel accumulation in neurons is **not** driven by residual VLP in the media. Replacing the media after 2 days post-transduction (as is required for iPSCs) did not significantly affect neuron editing efficiency at 4 days post-transduction. Notable because the steepest increase in indels in neurons typically occurs over the first 4 days post-transduction. Unpaired t test, ns = not significant. 6 replicate wells per condition, transduced in parallel. Dose: 1 μL VLP (FMLV) per 100 μL media. **c)** ABE-VLPs confirm that the slow indel accumulation we observed is **not** simply a product of “deficient” VLP delivery to neurons. When the same HIV VLPs are used to deliver adenine base editors (ABE8e) instead of Cas9, neurons can match and even exceed the editing efficiency of iPSCs, within only 3 days post-transduction. CRISPResso2 analysis of amplicon-NGS. Error bars show SEM; 3 replicate wells per condition, transduced in parallel. Dose: 4 μL VLP (HIV) per 100 μL media – but these VLPs were half as concentrated as normal, since VLPs were harvested from 3 10 cm plates per batch instead of 6 but still resuspended to the same volume. Therefore, equivalent to a 2 μL VLP dose from normal batches. ABE-VLP cloning protocol is described in the fourth tab of Supplementary Data 4.

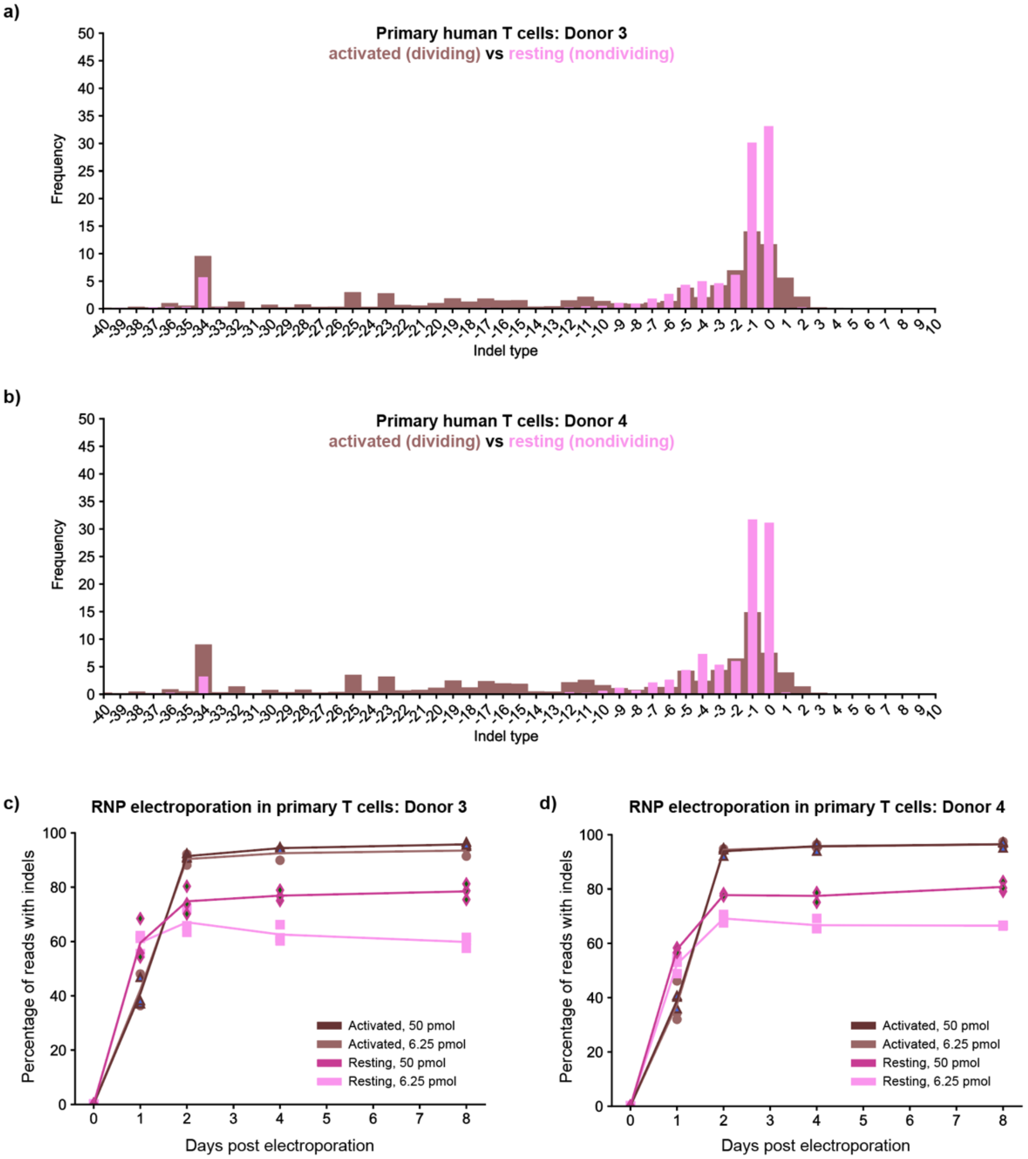

Supplementary Figure 10: Primary human T cells (with Cas9-RNP electroporation) reproduce some, but not all, of our findings from VLP-treated neurons.

a-b) Primary human T cells in the activated (dividing) state, compared to the resting (nondividing) state, reproduce the differences in prevalence of MMEJ-like vs NHEJ-like indels observed in iPSCs (dividing) vs neurons (nondividing). This is a second model of genetically identical dividing vs nondividing cells, but with Cas9 RNP delivered by electroporation instead of VLP. Note, however, that for this sgRNA (B2Mg1) resting T cells appear intrinsically far more deletion-biased (almost no insertions), compared to neurons. **c-d)** Indels do **not** accumulate for notably longer in Cas9-electroporated nondividing vs dividing T cells – unlike the dramatic differences observed in VLP-treated neurons vs iPSCs. CRISPResso2 analysis of amplicon-NGS.

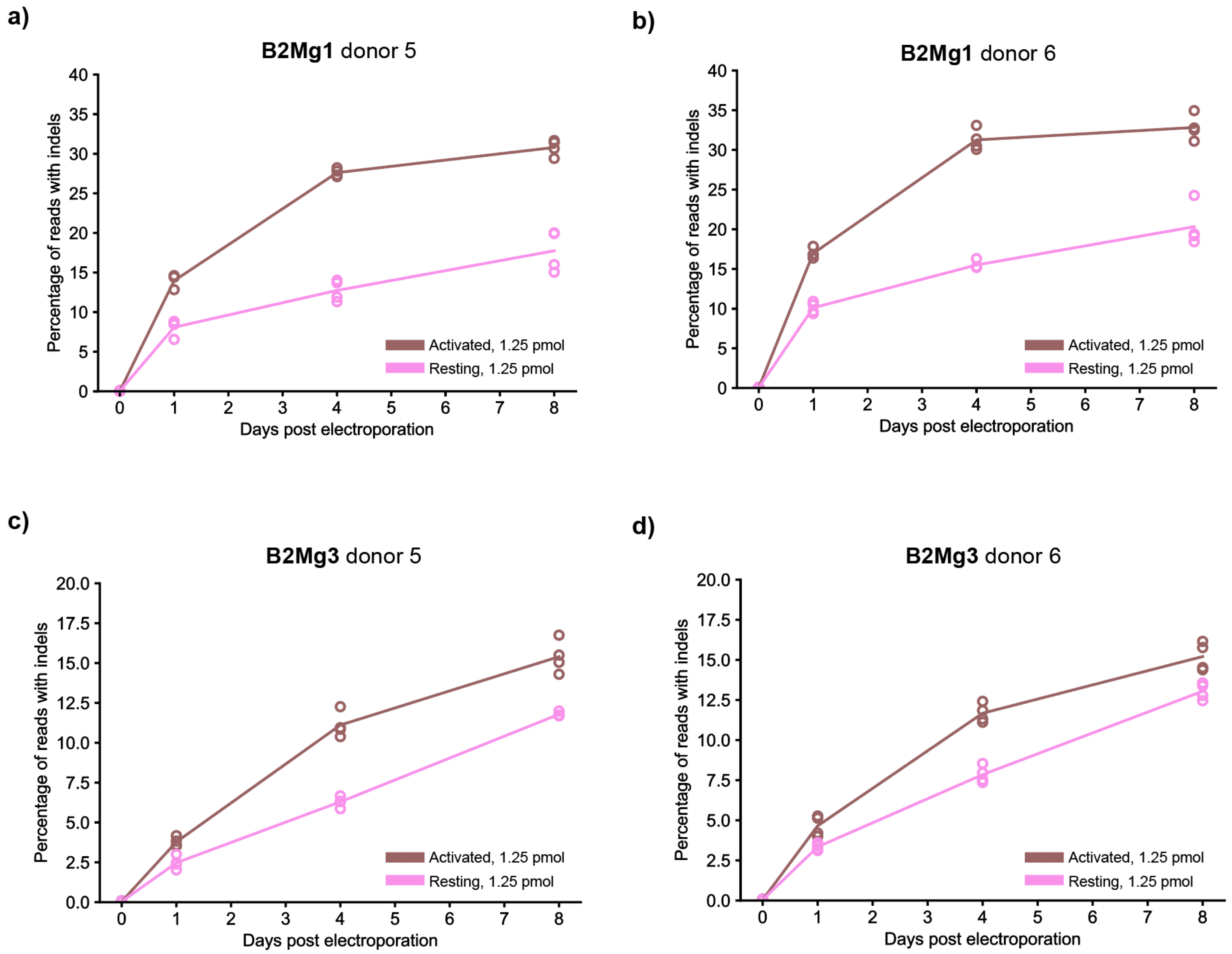

Supplementary Figure 11: The time course of indel accumulation is *not* dramatically different between nondividing and dividing primary human T cells, following Cas9 RNP electroporation.

a-d) Even with a lower Cas9 RNP dose, two independent sgRNAs, and two new human donors: Cas9-electroporated resting vs activated primary T cells do not reproduce the dramatic differences in indel kinetics observed in VLP-treated neurons/cardiomyocytes vs iPSCs. The prolonged accumulation of indels observed in postmitotic neurons and cardiomyocytes may therefore be dependent on cell type, and/or influenced by VLP delivery kinetics. CRISPResso2 analysis of amplicon-NGS.

a)

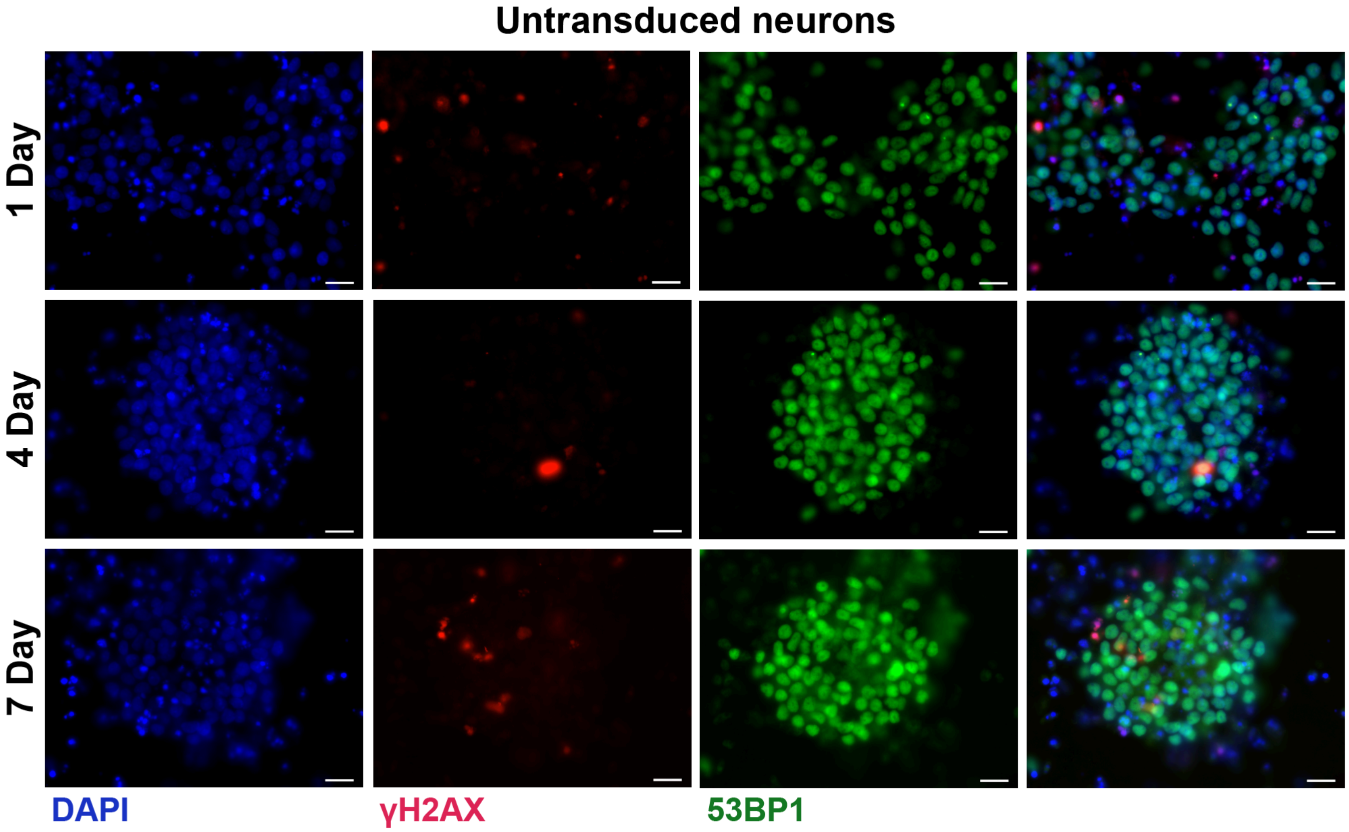

b)

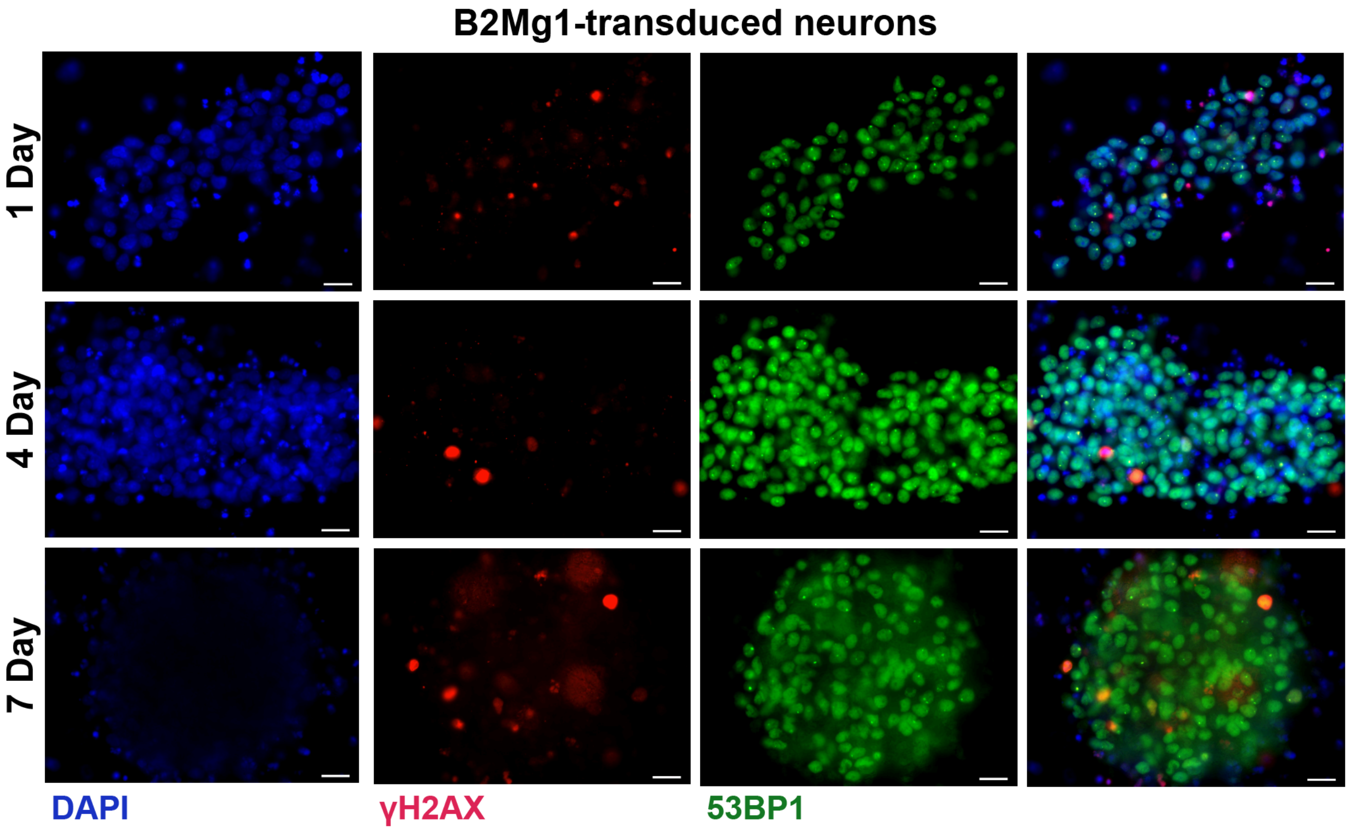

(continued on next page)

(continued from previous page)

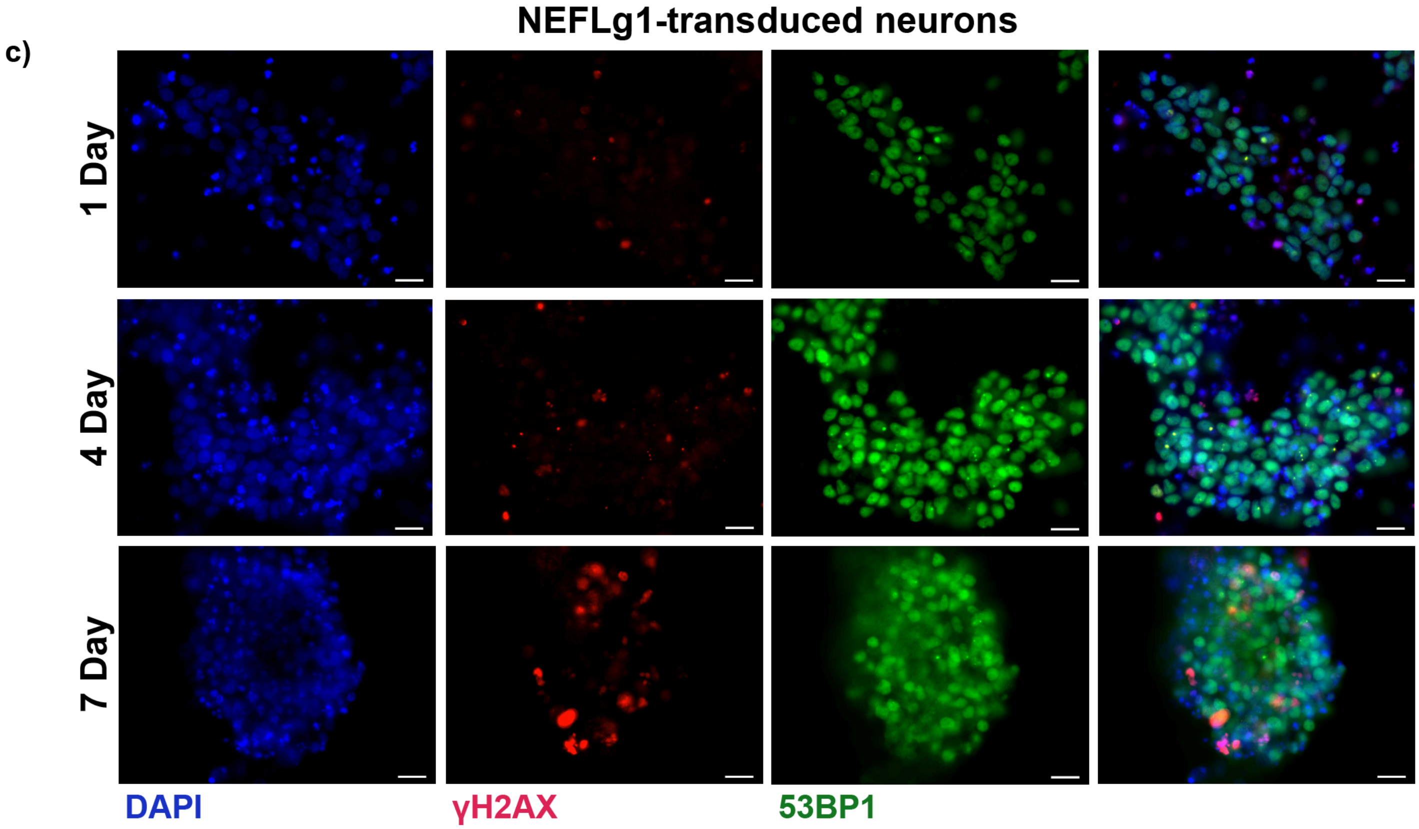

Supplementary Figure 12: Cas9-induced DSB repair signals persist in neurons for at least one week post-transduction.

a-c) DSB repair markers over time in untransduced (a), B2Mg1-transduced (b), and NEFLg1-transduced (c) neurons. DSBs are co-labeled by ICC markers γH2AX (red) and 53BP1 (green). Dose: 1 µL FMLV VLP per 100 µL media. Neurons were fixed at 1,4, or 7 days post-transduction as labeled. One representative image from each condition is shown. Transduction was 2 weeks into differentiation. Scale bar is 20 µm. Same experiment as Figure 2d-e, but now showing unmerged panels individually, and including additional conditions (timepoints, sgRNAs). Therefore, the merged panels for untransduced and B2Mg1-transduced at 1 day and 7 days are the same as in Figure 2d-e, but uncropped here.

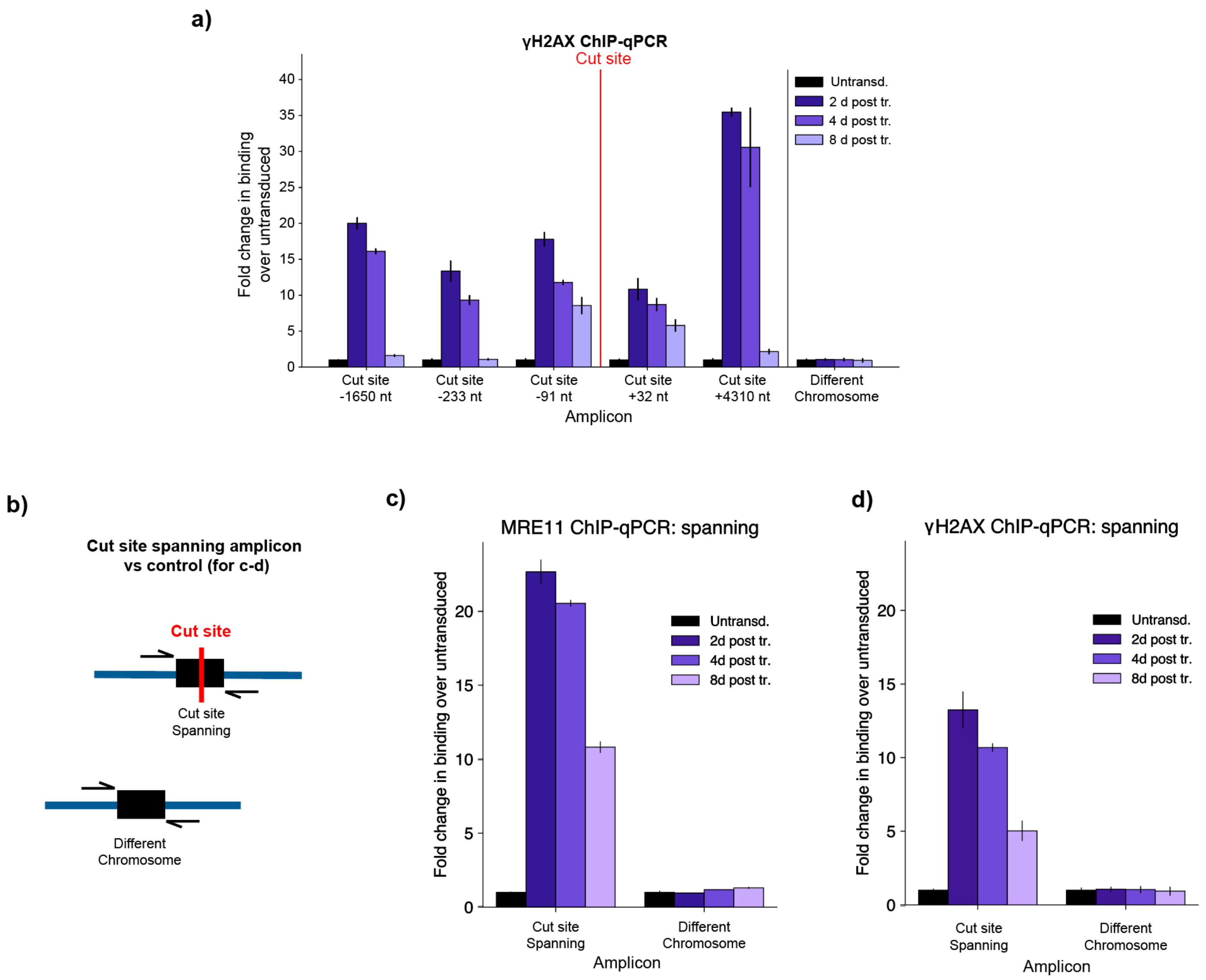

Supplementary Figure 13: DSB repair signals remain detectable at the cut site for at least 8 days post-transduction.

a) ChIP-qPCR for γH2AX binding at various distances from the cut site over time. Same conditions as Figure 2f, but with γH2AX antibody instead of Mre11. **b)** Schematic illustrating our strategy to detect cut-and-resealed loci by using a ChIP-qPCR amplicon that spans across the cut site. Repair protein binding suggests that the locus had been cut, and successful PCR amplification suggests that the cut has since been resealed. Note: however, it remains ambiguous whether these loci were sealed with or without an indel. **c-d)** Some loci have been resealed as early as 2 days post-transduction. ChIP-qPCR using the spanning amplicon to detect cut-and-resealed loci, with both Mre11 (c) and gH2AX (d). Same procedures as Figure 2f, but using different amplicons (cut site spanning, and different chromosome control).

a)

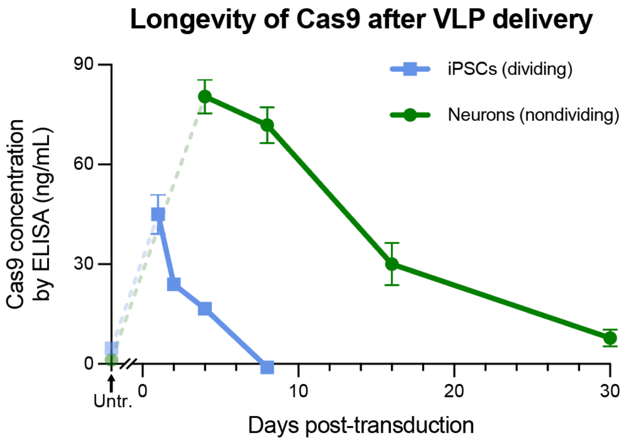

b)

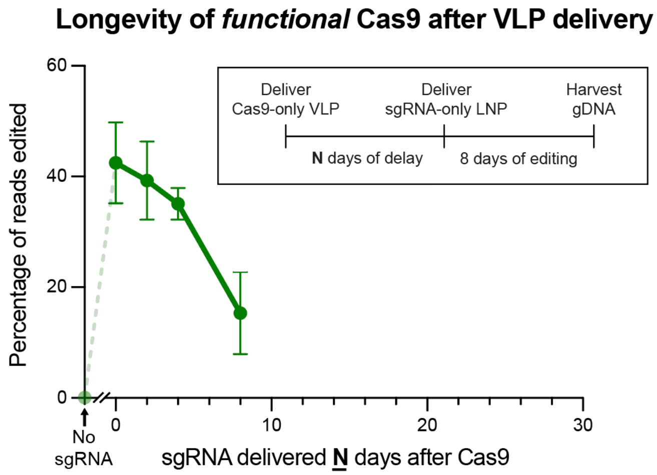

Supplementary Figure 14: Cas9 protein is unexpectedly long-lived in neurons following VLP delivery.
a) Cas9 protein remains detectable in neurons for at least 30 days after VLP transduction. Dose: 1 µL FMLV VLP (B2Mg1) per 100 µL media. Neurons were transduced at day 31 of differentiation and lysed at each timepoint using Whole Cell Extraction Buffer with 1000x Protease Inhibitor Cocktail and 1000x DTT (Epigentek, #OP-0003-100). Cas9 protein concentration in whole cell lysate was then measured by ELISA (Cell Biolabs, #PRB-5079). 6 replicate wells per condition were transfected in parallel in 96-well format. To maximize input, all 6 wells' lysates were then pooled together, and split into 2 replicate ELISA reactions per condition. Error bars show range of these 2 replicate ELISA reactions. **b)** Functional Cas9 remains present in neurons for at least 8 days after VLP transduction. 2 µL FMLV VLP (Cas9 only, no sgRNA) was delivered per 100 µL media, followed by 125 ng of B2Mg1 sgRNA (via F16-15 lipid nanoparticle) delivered at various timepoints later. 8 days after sgRNA delivery, gDNA was harvested and indels were quantified via CRISPResso2 analysis of amplicon-NGS. 6 replicate wells per condition transfected in parallel; error bars show standard deviation.

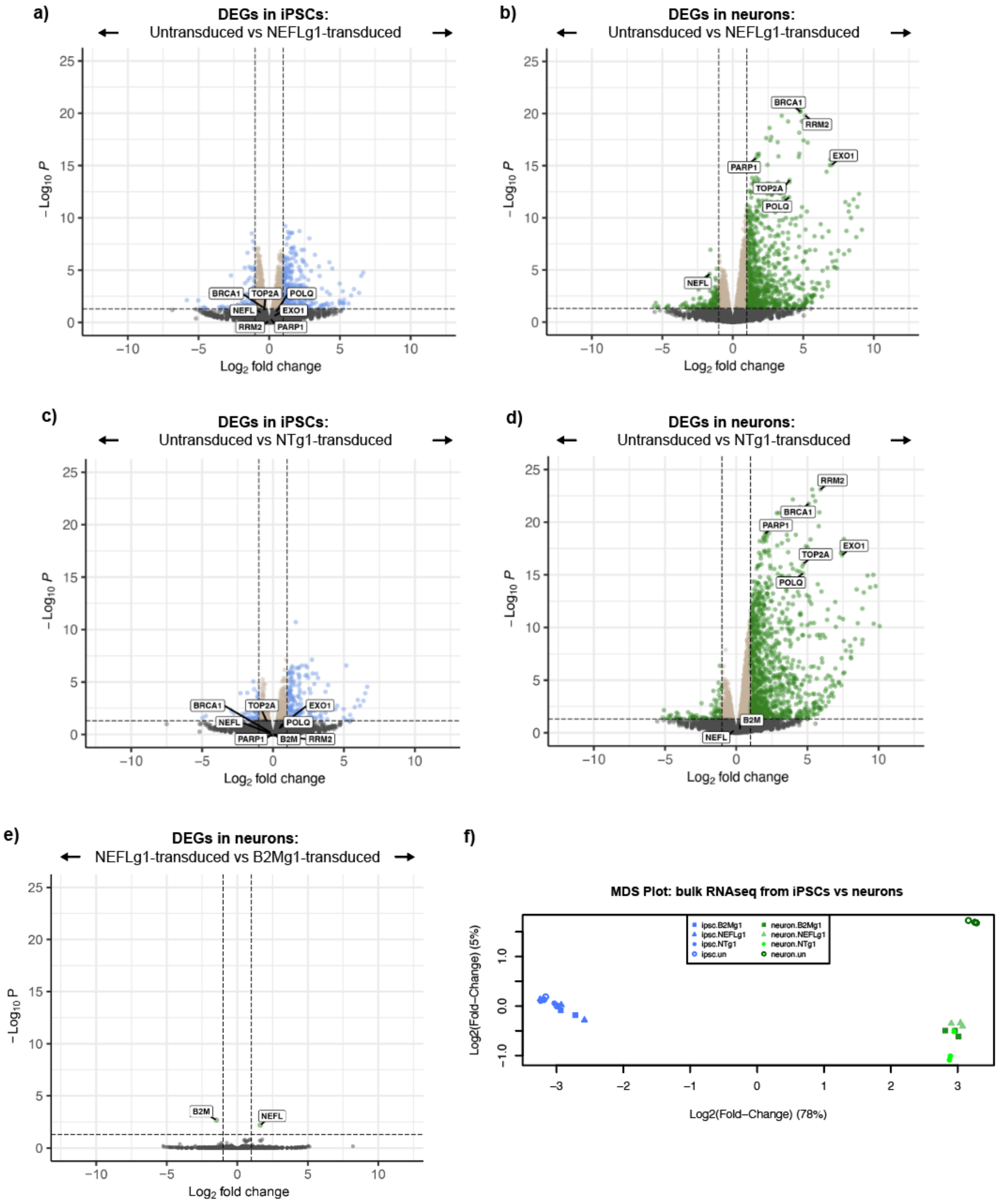

Supplementary Figure 15: Neuronal transcriptional response to Cas9-VLP is very consistent across three different sgRNAs.

a-d) The neuron-specific transcriptional response to Cas9-VLPs was replicated by two additional sgRNAs, NEFLg1 (a-b) and NTg1 (c-d), besides B2Mg1 shown in Figure 3a-b. Neurons have more DEGs overall upon transduction, and the most

significant of these DEGs are enriched for DNA repair genes. Same parameters as Figure 3a-b, but with different sgRNAs. Note: *NEFL* is not expressed in iPSCs, so its expression is not expected to decrease upon NEFLg1 editing in iPSCs. **e)** The only two DEGs between B2Mg1-edited and NEFLg1-edited neurons are *B2M* and *NEFL* respectively. This reinforces the consistency of the neuronal transcriptional response across different sgRNAs. **f)** Multidimensional scaling (MDS) plot showing unbiased clustering of the various RNAseq samples. While VLP-transduced iPSC samples clustered together with untransduced iPSCs, VLP-transduced neurons clustered separately from untransduced neurons. **Note**: in this experiment, NTg1 puzzlingly elicited a similar (or greater) amplitude of this neuronal response compared to the targeting sgRNAs. In Supplementary Figure 19 we show a followup experiment with additional controls, in which targeting Cas9-VLPs elicited a significantly stronger response than both non-targeting VLPs or dCas9-VLPs, as expected.

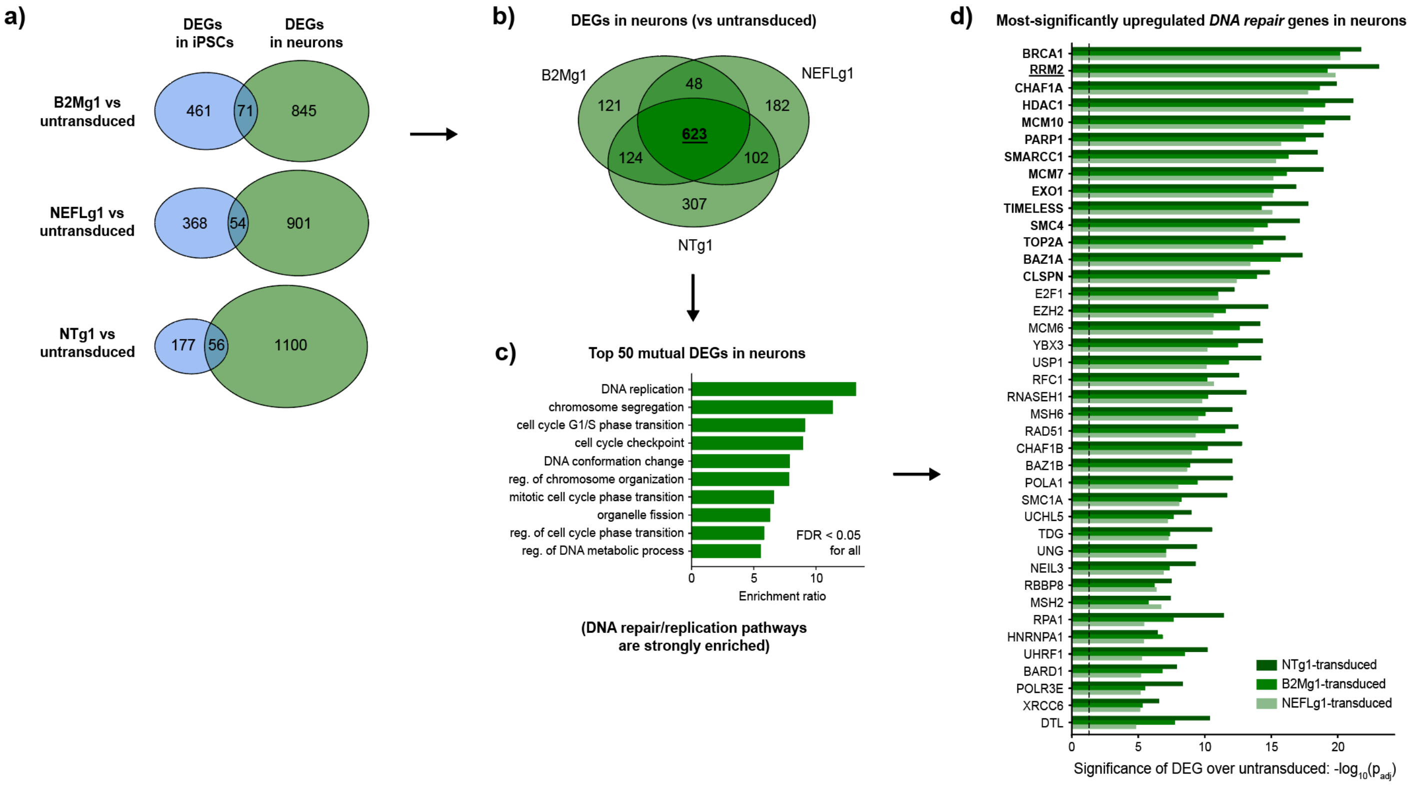

Supplementary Figure 16: Neurons exhibit a distinct transcriptional response to Cas9-VLPs, upregulating unexpected genes canonically associated with DNA repair/replication.

a) Transduced neurons consistently have more DEGs than transduced iPSCs for 3 different sgRNAs, and <10% of DEGs are shared between the cell types. **b)** Over 75% of the DEGs in either B2Mg1- or NEFLg1-transduced neurons are shared with NTg1-transduced neurons. **c)** The most significantly altered DEGs in transduced neurons are highly enriched for DNA repair factors. **d)** Transduced neurons significantly upregulate many DNA repair genes, including factors canonically associated with replication. Top 40 DNA repair DEGs are shown, rank-ordered by averaging the adjusted p-values from each transduced condition. Bold denotes repair genes ranked in the top 50 DEGs genome-wide. **Note**: in this experiment, NTg1 puzzlingly elicited a similar (or greater) amplitude of this neuronal response compared to the targeting sgRNAs. In Supplementary Figure 19 we show a followup experiment with additional controls, in which targeting Cas9-VLPs elicited a significantly stronger response than both non-targeting VLPs or dCas9-VLPs, as expected.

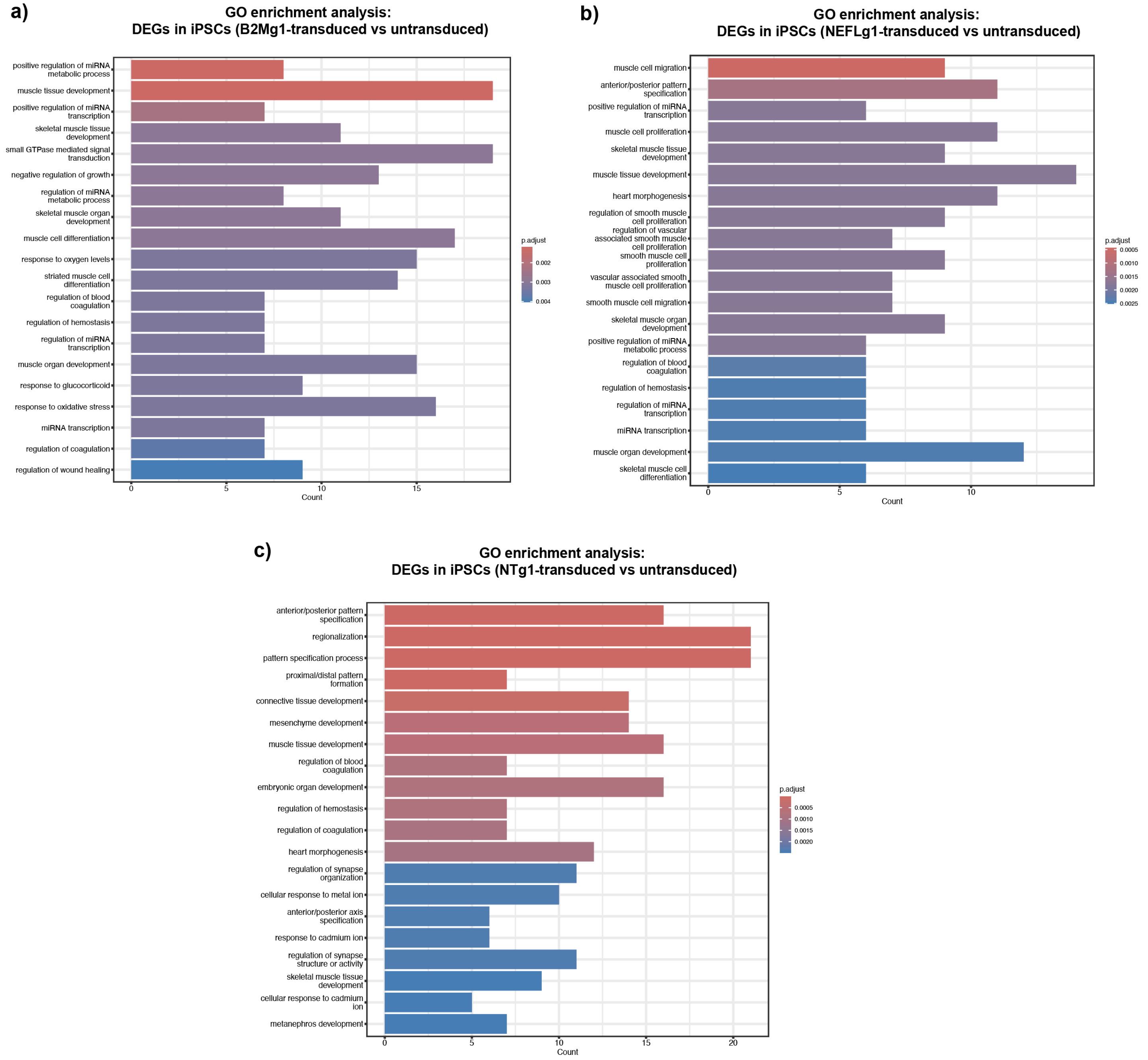

Supplementary Figure 17: DNA repair genes are not enriched in the DEGs of transduced iPSCs.
a-c) Gene ontology (GO) analysis shows no enrichment for DNA repair genes in the DEGs of B2Mg1-transduced (a), NEFLg1-transduced (b), or NTg1-transduced (c) iPSCs, relative to untransduced iPSCs. Showing the top 20 GO terms in each comparison. Bar length indicates number of DEGs that fall into each GO category. Color indicates significance of adjusted p-value.

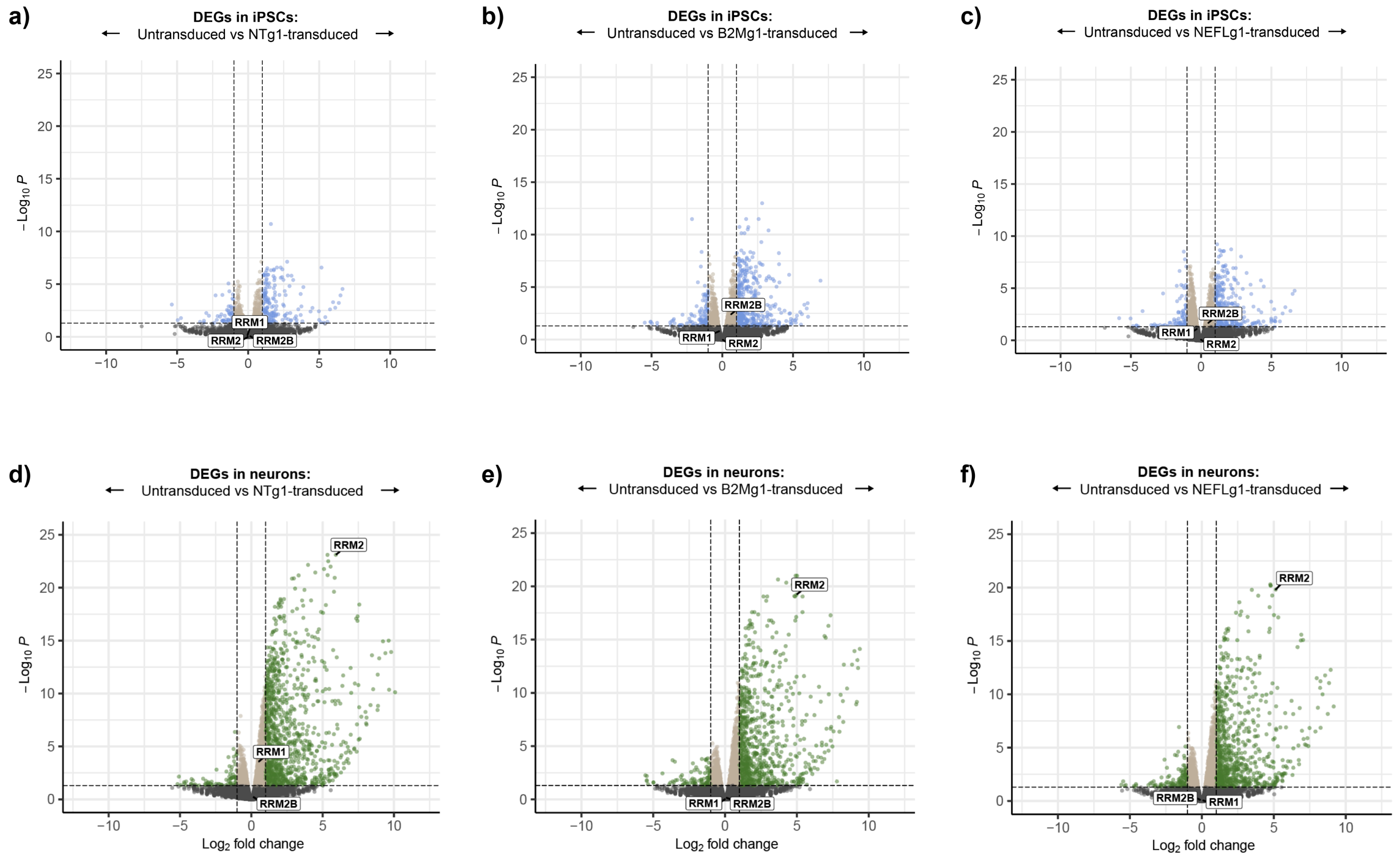

Supplementary Figure 18: Transcriptional response of RNR subunits in neurons compared to iPSCs.
a-c) In iPSCs, non-targeting Cas9 (a) does not affect transcription of any RNR subunits. However, both of the cutting Cas9-VLPs (b-c) significantly induce transcription of *RRM2B*, the canonically DSB-responsive subunit of RNR. The other two subunits of RNR are unaffected in iPSCs. **d-f)** In neurons, the canonically S-phase-restricted *RRM2* is one of the most significantly upregulated genes in all 3 transduced conditions, including with non-targeting Cas9.

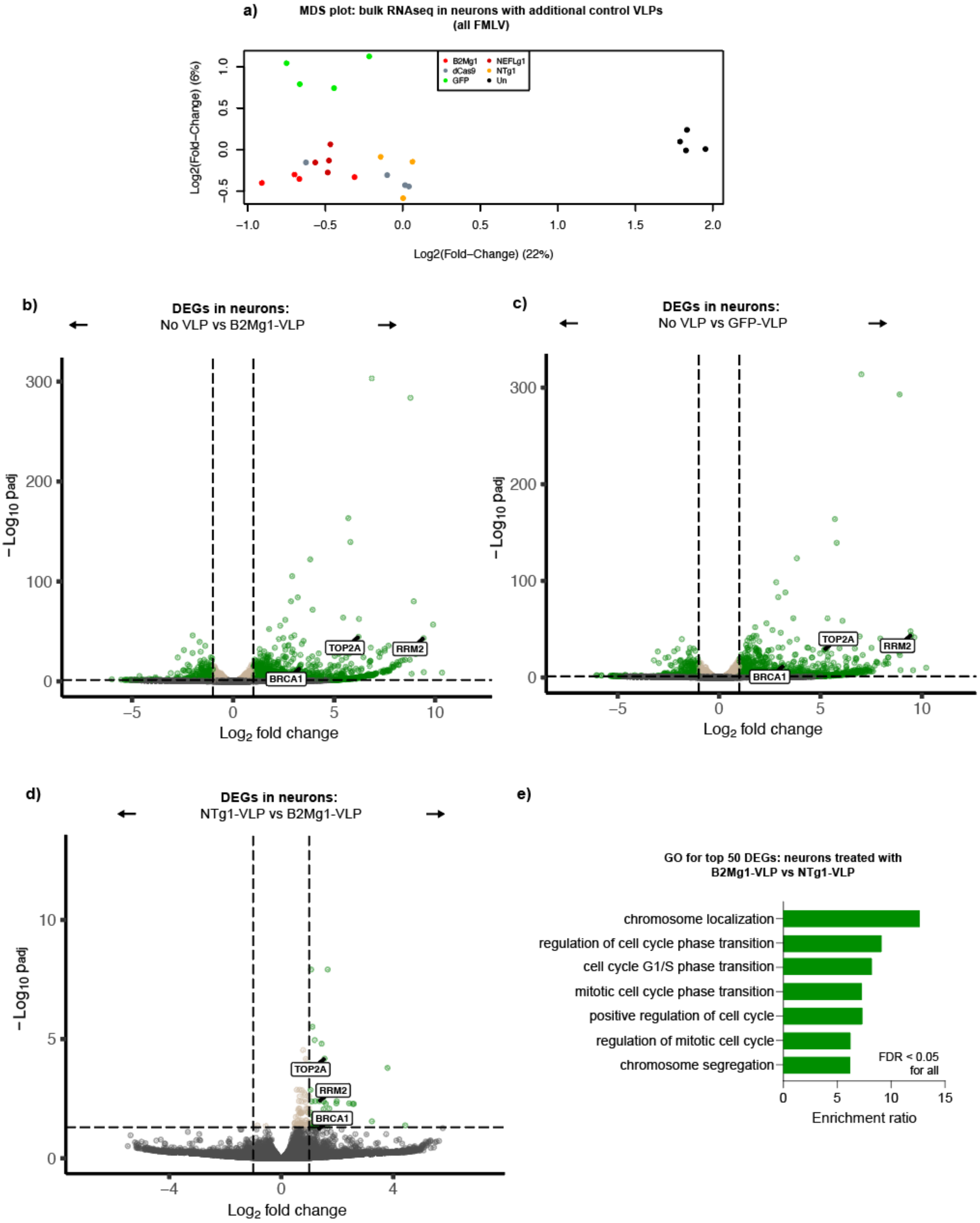

(continued on next page)

(continued from previous page)

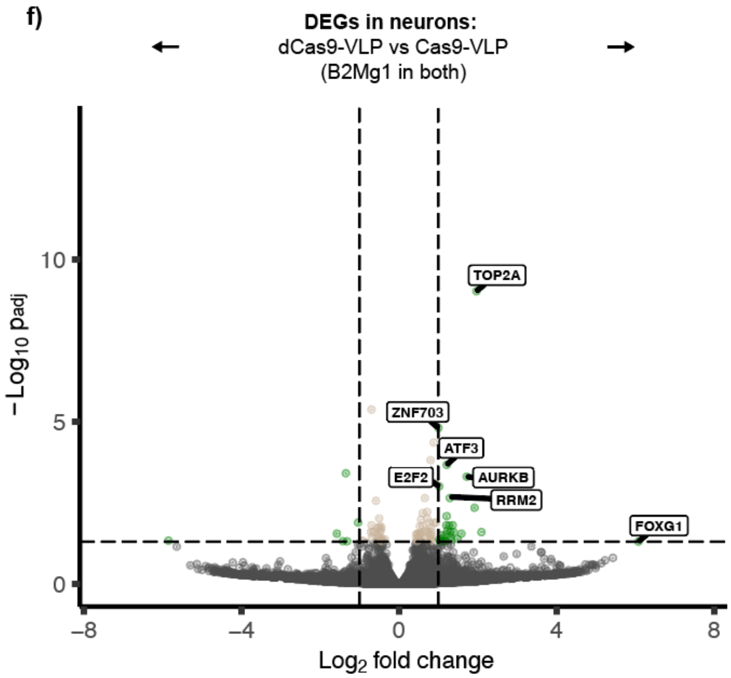

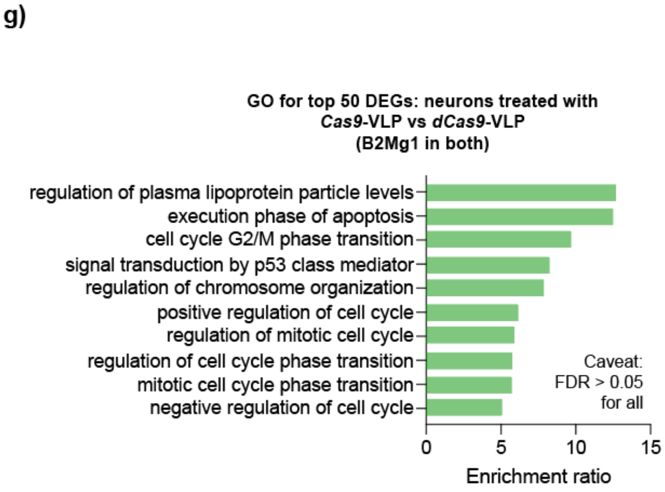

Supplementary Figure 19: The neuronal transcriptional response to VLPs is amplified by, but not specific to, Cas9-induced DSBs.

a) Multidimensional scaling (MDS) plot showing unbiased clustering of additional RNAseq control samples: untransduced neurons, or neurons transduced with FMLV VLPs containing Cas9-B2Mg1, Cas9-NEFLg1, Cas9-NTg1, dCas9-B2Mg1, or GFP. Untransduced neurons cluster separately from all transduced neuron samples, but neuron samples treated with GFP-VLPs cluster separately from those treated with Cas9-VLPs. **b-c)** RRM2 is upregulated by neurons in response to both B2Mg1-VLPs and GFP-VLPs. Therefore, a large component of the neuronal transcriptional response is likely induced by VLPs themselves. **d-e)** Neuronal upregulation of DNA repair/replication genes, including RRM2, is significantly higher in response to Cas9-B2Mg1 VLPs compared to Cas9-NTg1 VLPs. Therefore, some component of this response is dependent on (or amplified by) the presence of DSBs. **f-g)** Neuronal upregulation of DNA repair/replication genes, including RRM2, is significantly higher in response to Cas9-B2Mg1 VLPs compared to dCas9-B2Mg1 VLPs. Therefore, some component of this response is again dependent on (or amplified by) the presence of DSBs. For these FMLV VLP RNAseq results, dose: 2 μL FMLV VLP per 20,000 cells.

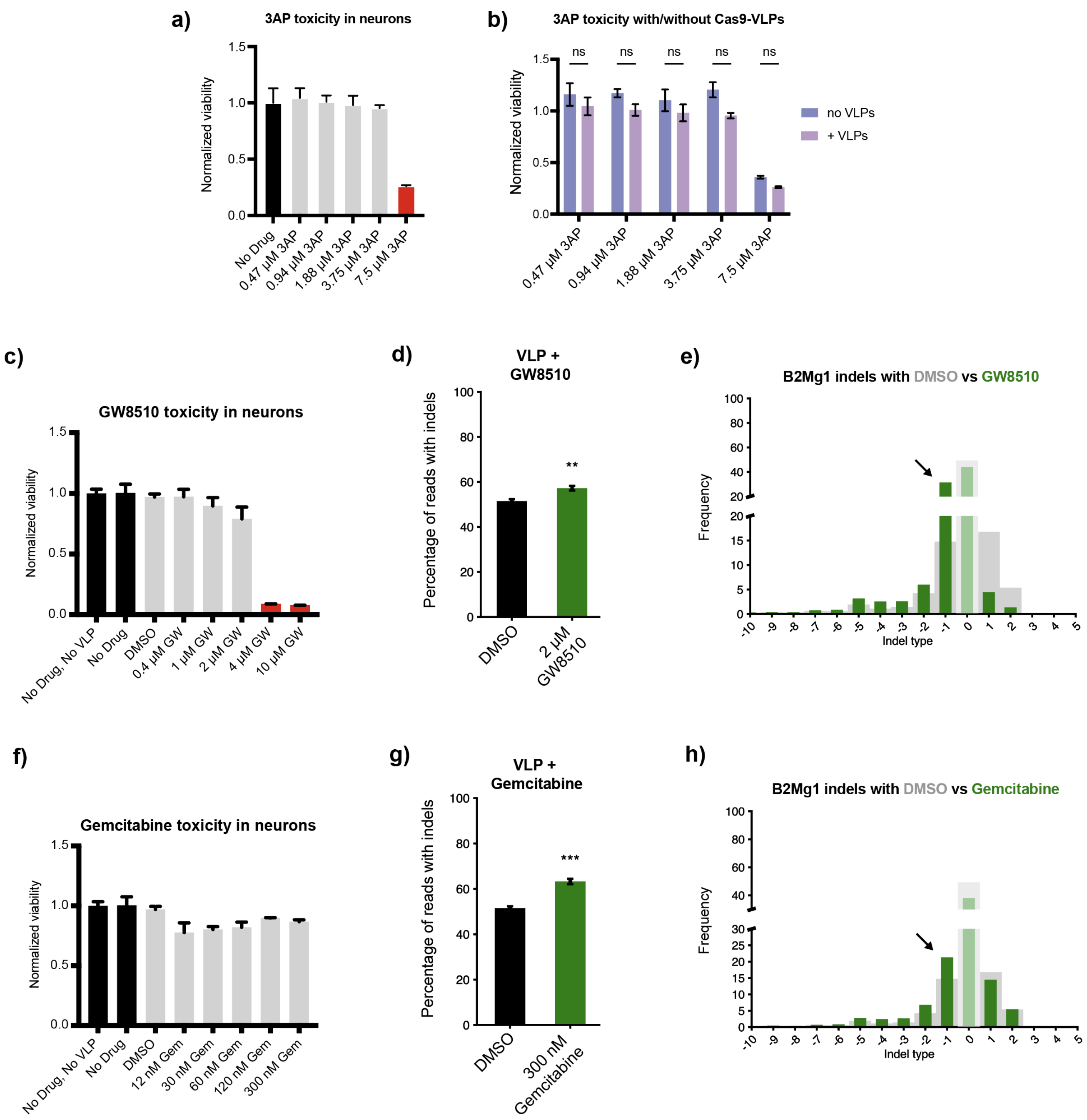

Supplementary Figure 20: Inhibiting RNR alters editing outcomes in neurons.
a) Toxicity of escalating doses of RRM2 inhibitor 3AP in neurons. Maximum tolerable dose was 3.75 µM. For a/c/f: Tolerability threshold was arbitrarily set to 0.75 or above, corresponding to less than a 25% reduction in viability. PrestoBlue viability assay at 8 days post-transduction, normalized to age-matched untreated neurons on the same plate. 3 replicate wells per condition, treated in parallel; error bars show SEM. **b)** Toxicity of escalating 3AP doses in neurons with or without Cas9-VLPs. Optimal 3.75 µM dose remains nontoxic even with Cas9-VLPs (1 µL FMLV) inducing DNA damage. Error bars show SEM. Two-factor ANOVA; ns = not significant ($p>0.05$). **c)** Toxicity of escalating doses of RRM2 inhibitor GW8510 in neurons, alongside Cas9-VLP treatment. Maximum tolerable dose was 2 µM. **d-e)** GW8510 co-treatment of B2Mg1-edited neurons increases indels overall (d), boosting deletions specifically, and roughly doubles the frequency of single-base deletions (e). Replicated the effects of RRM2 inhibitor 3AP from Figure 3g-h. Dose: 1 µL FMLV VLP per 100 µL media, and maximum tolerable dose of GW8510. Indels measured 8 days post-transduction. For d, error bars show SEM. One-Factor ANOVA, ** $p<0.005$. For d-e, 6 replicate wells per condition treated in parallel. **f)** Toxicity of escalating doses of RRM1 inhibitor gemcitabine in neurons, alongside Cas9-VLP treatment. Maximum tested dose was

300 nM, and still tolerable. **g-h)** Gemcitabine co-treatment of B2Mg1-edited neurons increases indels overall (g), boosting deletions specifically (h). Replicated the effect of RRM2 inhibitor 3AP from Figure 3g-h. Dose: 1 µL FMLV VLP per 100 µL media, and maximum tolerable dose of gemcitabine. Indels measured 8 days post-transduction. For g, error bars show SEM. One-Factor ANOVA, *** $p<0.0005$. For g-h, 6 replicate wells per condition treated in parallel.

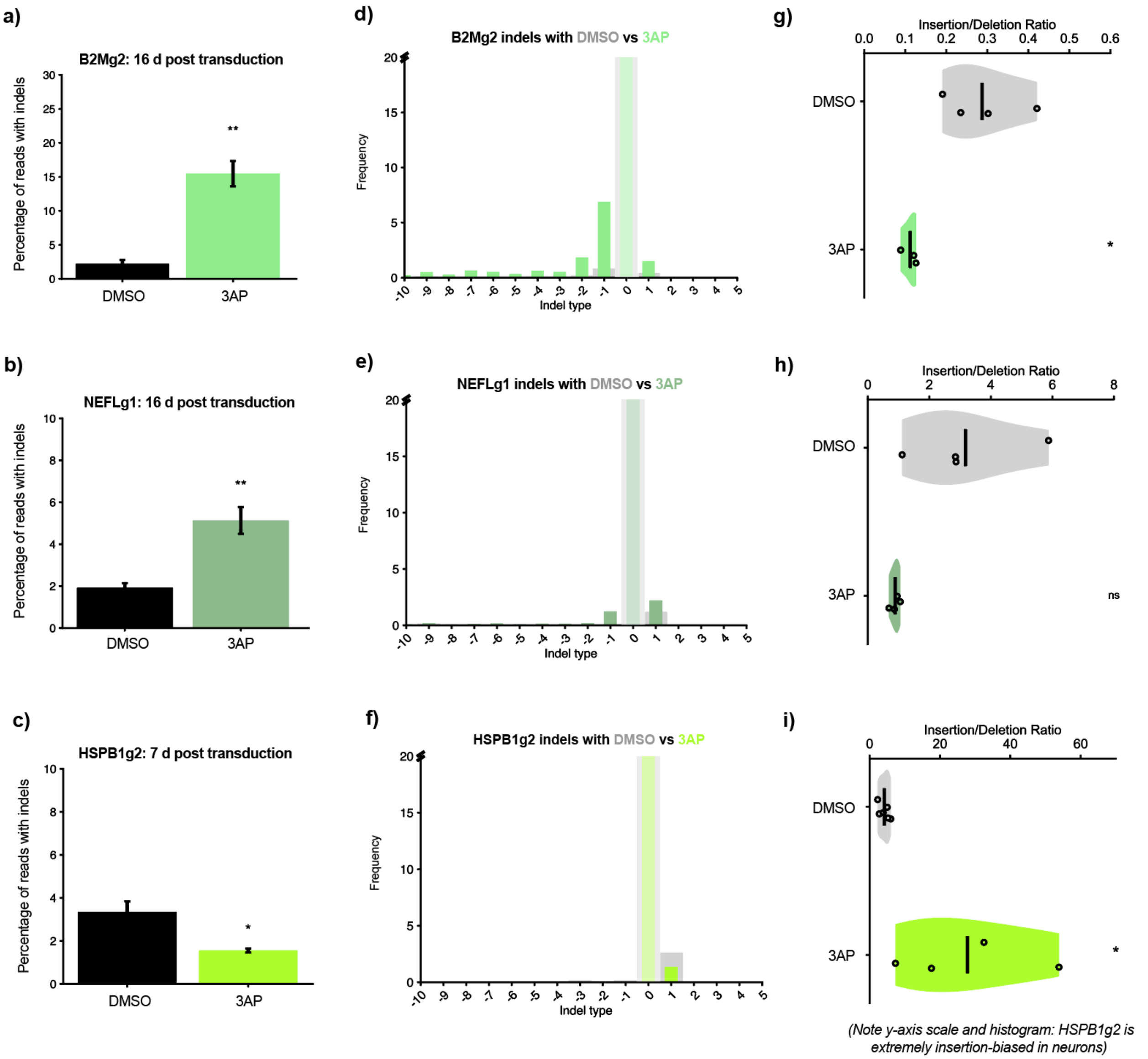

Supplementary Figure 21: RNR inhibition affects neuron editing outcomes in an sgRNA-dependent manner. **a-c)** 3AP treatment significantly increases total indels for B2Mg2 (a) and NEFLg1 (b), but significantly decreases total indels for the intrinsically insertion-biased HSPB1g2 (c). **d-f)** The sgRNA-dependent effects of 3AP seen in a-c are consistent with their underlying indel distributions, as HSPB1g2 appears relatively impermissible to deletions. **g-i)** For B2Mg2 and NEFLg1, but not for HSPB1g2, 3AP treatment shifts indels from insertions toward deletions – as it does for B2Mg1 (Figure 3). For all: 6 replicate wells per condition, transduced in parallel. 1 µL FMLV VLP per 20,000 cells in 100 µL media. One-Factor ANOVA, * $p<0.05$, ** $p<0.005$, ns = not significant.

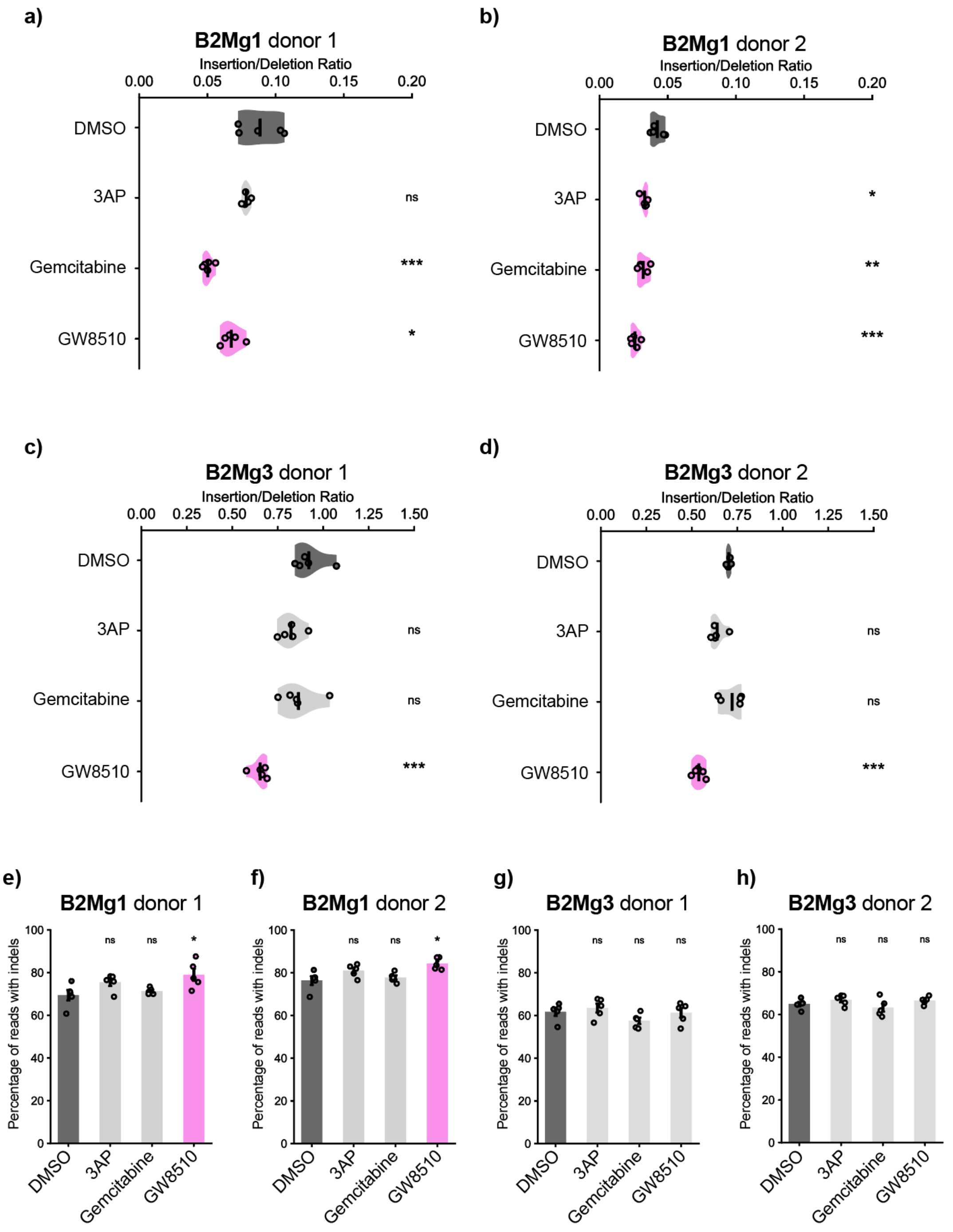

Supplementary Figure 22: Cas9-electroporated nondividing primary T cells reproduce the effects of RRM2 inhibition observed in VLP-treated neurons.

a-d) RRM2 inhibition shifts indels from insertions toward deletions in Cas9-electroporated resting (nondividing) primary T cells: from 2 independent human donors, with 2 independent sgRNAs. This matches the effect of RRM2 inhibition on editing outcomes in neurons. **e-h)** RRM2 inhibitor GW8510 can increase total indel efficiency in Cas9-electroporated resting (nondividing) primary T cells: in a sgRNA-dependent manner, consistently for two independent human donors. Total indels increased for B2Mg1, but not for the more insertion-biased B2Mg3. This matches the sgRNA-dependent effects of RRM2 inhibition on neurons: where RRM2 inhibition increased total indels for sgRNAs including B2Mg1, but not for the more insertion-biased HSPB1g2. For all: CRISPResso2 analysis of amplicon-NGS, 4 days post-electroporation with 6.25 pmol of Cas9 RNP per nucleofection well.

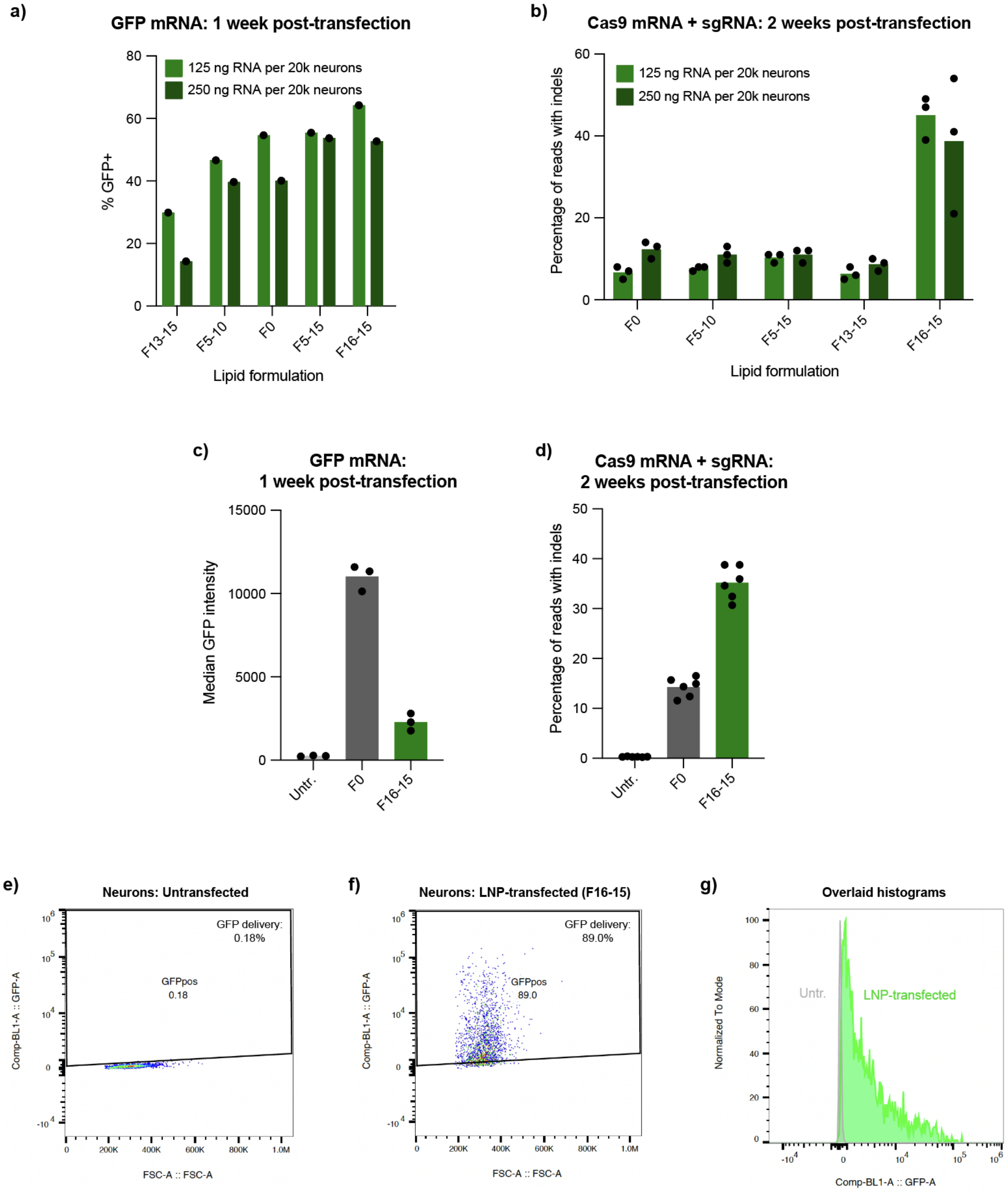

Supplementary Figure 23: F16-15 LNP formulation allows efficient RNA delivery to neurons.
a) LNP formulation F16-15 effectively delivers GFP mRNA to human iPSC-derived neurons. **b)** LNP formulation F16-15 effectively delivers Cas9 mRNA and sgRNA into iPSC-derived neurons. Data from Figure 4a, but with both mRNA doses shown here: 125 ng and 250 ng per 100 µL media. **c-d)** While F0 (D-Lin-MC3) appears to outperform F16-15 for median GFP delivery per neuron (c), F16-15 outperforms F0 for Cas9+sgRNA delivery (d). The optimal LNP formulation for a given cell type appears to depend on the type of cargo being delivered. **e-g)** F16-15 LNPs deliver GFP mRNA to neurons with a maximum efficiency of almost 90%. One replicate shown, representative of the higher end of F16-15 delivery efficiency. For a/c/e/f/g: GFP signal measured by flow cytometry. For b: indels measured by Synthego ICEv2. For d: indels measured by CRISPResso2 analysis of amplicon-NGS. For c-g: 125 ng of total RNA per 100 µL media.

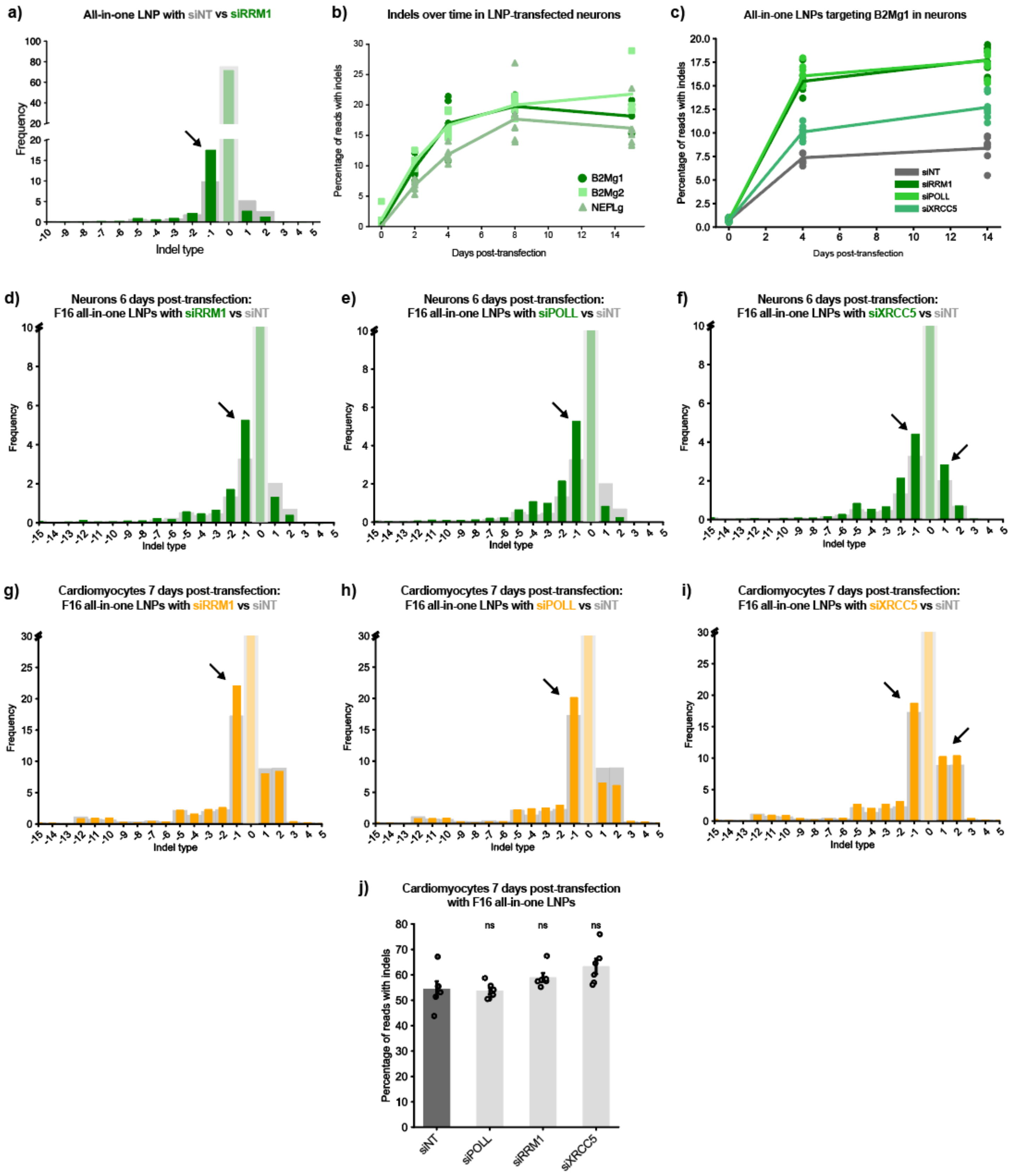

Supplementary Figure 24: All-in-one LNPs reveal consistent effects of DNA repair factors on CRISPR editing outcomes in postmitotic neurons and cardiomyocytes.

a) All-in-one LNPs which knock down RRM1 during editing phenocopy small molecule inhibition of RRM1/2: shifting editing outcomes from insertions toward deletions. Two weeks post-transfection (B2Mg1). For a-j: CRISPResso2 analysis of amplicon-NGS after F16-15 LNP transfection, with 125 ng total RNA per 100 µL media. **b)** Multiple sgRNAs show days-long accumulation of neuron indels following LNP transfection. Individual points represent 6 replicate wells per condition

transfected in parallel (some obscured by overlap). Curves connect means at each timepoint. **c)** When incorporated into all-in-one LNPs, the 3 “hit” siRNAs increase total indels in neurons at both early and late timepoints (relative to siNT). **d-f)** In neurons, all-in-one LNPs knocking down RRM1 or POLL shift indels from insertions toward deletions. Knocking down XRCC5 increases both insertions and deletions. **g-i)** In cardiomyocytes, all-in-one LNPs knocking down RRM1 or POLL shift indels from insertions toward deletions. Knocking down XRCC5 increases both insertions and deletions. **j)** In cardiomyocytes, the 3 “hit” siRNAs did not significantly increase *total* indels at 7 days post-transfection. For g-j: cardiomyocytes were generated from WTC background iPSCs using the protocol described in Perez-Bermejo et al, Sci Transl Med, 2021 (PMCID: PMC8128284), then transfected at day 30+ of differentiation, after lactate purification to select for postmitotic CMs.
