# Supplementary material for: Characterizing and controlling CRISPR repair outcomes in nondividing human cells
Source: Nat Commun. 2025 Nov 17;16:9883. doi: 10.1038/s41467-025-66058-3 (PMC12623481; doi:10.1038/s41467-025-66058-3)
Supplement: Supplementary file 2 — Description of Additional Supplementary Files [file 41467_2025_66058_MOESM2_ESM.pdf]

**Description of Additional Supplementary Files**

File Name: Supplementary Data 1
Description: Counts table for initial RNAseq

File Name: Supplementary Data 2
Description: Counts table for second RNAseq with additional control

File Name: Supplementary Data 3
Description: NGN2 neuron differentiation protocol and calculation sheet

File Name: Supplementary Data 4
Description: FMLV VLP production protocol and calculation sheet

File Name: Supplementary Data 5
Description: sgRNA modification template for LNPs

File Name: Supplementary Data 6
Description: Sequences of sgRNAs and NGS primers

File Name: Supplementary Data 7
Description: Drugs and concentrations

File Name: Supplementary Data 8
Description: ChIP-qPCR primers and antibodies
