# Supplementary material for: Characterizing and controlling CRISPR repair outcomes in nondividing human cells
Source: Nat Commun. 2025 Nov 17;16:9883. doi: 10.1038/s41467-025-66058-3 (PMC12623481; doi:10.1038/s41467-025-66058-3)
Supplement: Supplementary file 11 — Reporting Summary [file 41467_2025_66058_MOESM11_ESM.pdf]

nature portfolio

Corresponding author(s): Bruce R Conklin

Last updated by author(s): Sep 24, 2025

Reporting Summary

Nature Portfolio wishes to improve the reproducibility of the work that we publish. This form provides structure for consistency and transparency in reporting. For further information on Nature Portfolio policies, see our Editorial Policies and the Editorial Policy Checklist.

Statistics

For all statistical analyses, confirm that the following items are present in the figure legend, table legend, main text, or Methods section.

n/a	Confirmed	
☐	☒	The exact sample size (*n*) for each experimental group/condition, given as a discrete number and unit of measurement
☐	☒	A statement on whether measurements were taken from distinct samples or whether the same sample was measured repeatedly
☐	☒	The statistical test(s) used AND whether they are one- or two-sided<br>*Only common tests should be described solely by name; describe more complex techniques in the Methods section.*
☐	☒	A description of all covariates tested
☐	☒	A description of any assumptions or corrections, such as tests of normality and adjustment for multiple comparisons
☐	☒	A full description of the statistical parameters including central tendency (e.g. means) or other basic estimates (e.g. regression coefficient) AND variation (e.g. standard deviation) or associated estimates of uncertainty (e.g. confidence intervals)
☐	☒	For null hypothesis testing, the test statistic (e.g. *F*, *t*, *r*) with confidence intervals, effect sizes, degrees of freedom and *P* value noted<br>*Give P values as exact values whenever suitable.*
☒	☐	For Bayesian analysis, information on the choice of priors and Markov chain Monte Carlo settings
☒	☐	For hierarchical and complex designs, identification of the appropriate level for tests and full reporting of outcomes
☒	☐	Estimates of effect sizes (e.g. Cohen's *d*, Pearson's *r*), indicating how they were calculated

Our web collection on statistics for biologists contains articles on many of the points above.

Software and code

Policy information about availability of computer code

Data collection	Data collection for this study did not require software; only data analysis required software.
Data analysis	Genome editing analysis was performed using CRISPResso v2, as described in figure legends and Methods. RNAseq analysis was performed using EdgeR, as described in Methods.

For manuscripts utilizing custom algorithms or software that are central to the research but not yet described in published literature, software must be made available to editors and reviewers. We strongly encourage code deposition in a community repository (e.g. GitHub). See the Nature Portfolio guidelines for submitting code & software for further information.

Data

Policy information about availability of data

All manuscripts must include a data availability statement. This statement should provide the following information, where applicable:

- Accession codes, unique identifiers, or web links for publicly available datasets
- A description of any restrictions on data availability
- For clinical datasets or third party data, please ensure that the statement adheres to our policy

Source data are provided with this paper. Raw and processed RNA sequencing files have been deposited in the NCBI Gene Expression Omnibus (GEO) under accession code GSE272812 [https://www.ncbi.nlm.nih.gov/geo/query/acc.cgi?acc=GSE272812] for the first set comparing iPSC and neuron samples, and under accession code GSE304183 [https://www.ncbi.nlm.nih.gov/geo/query/acc.cgi?acc=GSE304183] for the second set evaluating additional control VLPs in neurons.

Research involving human participants, their data, or biological material

Policy information about studies with human participants or human data. See also policy information about sex, gender (identity/presentation), and sexual orientation and race, ethnicity and racism.

Reporting on sex and gender	N/A; our research does not involve human participants or their data.
Reporting on race, ethnicity, or other socially relevant groupings	N/A; our research does not involve human participants or their data.
Population characteristics	N/A; our research does not involve human participants or their data.
Recruitment	N/A; our research does not involve human participants or their data.
Ethics oversight	N/A; our research does not involve human participants or their data.

Note that full information on the approval of the study protocol must also be provided in the manuscript.

Field-specific reporting

Please select the one below that is the best fit for your research. If you are not sure, read the appropriate sections before making your selection.

☒ Life sciences ☐ Behavioural & social sciences ☐ Ecological, evolutionary & environmental sciences

For a reference copy of the document with all sections, see nature.com/documents/nr-reporting-summary-flat.pdf

Life sciences study design

All studies must disclose on these points even when the disclosure is negative.

Sample size	Sample sizes of 3-8 replicates per condition (as denoted in figure legends) were chosen based on empirical assessment of variability and likelihood of sample loss due to eg sequencing quality cutoffs. For example, for straightforward assays such as staining, 3 replicates were sufficient. However, for assays such as NGS genome editing analysis, where certain replicates might be discarded prior to analysis due to factors such as excessive cell death causing insufficient gDNA yield, NGS PCR reactions working too inefficiently, or sequencing quality failing alignment cutoffs, more than 3 replicates were used for these assays, so that even after any such samples were lost, at least 3 samples would undoubtedly remain, allowing proper determination of mean, standard deviation, and statistical tests such as ANOVA.
Data exclusions	Exclusion criteria for genome editing NGS analysis were pre-determined: any samples with less than 1000 merged reads were excluded prior to CRISPResso analysis. Furthermore, in any experiments with less than 90% alignment across samples, a quality threshold was pre-determined to exclude any samples with less than 60% of reads aligning to the amplicon of interest.
Replication	Key experimental findings, namely the time course findings, were reproducibly observed by multiple different individuals performing the experiments themselves - with different sgRNAs, different batches of cells, different batches of VLP, etc. Furthermore, some such findings also replicated even between two completely different nondividing cell types (eg neurons and cardiomyocytes). Additionally, key DNA repair findings such as the influence of RNR on editing outcomes were replicated with 2 different forms of Cas9 delivery (VLP and LNP), and 4 different methods of RNR inhibition (3 different small molecule inhibitors, plus siRNA knockdown).
Randomization	Not relevant to our study; our study did not use human participants, organisms, or groups of primary samples.
Blinding	Blinding was not critical to our study, since we did not use human participants, organisms, or groups of primary samples. For these cell culture experiments, investigators collecting data were unable to be blinded to which condition was in each well, because all VLP transductions and LNP transfections were performed by hand, and therefore required knowledge of which reagents went into each well. However, after the gDNA samples were collected, the NGS library prep and sequencing itself was done by a blinded core service, which had no knowledge of the samples and conditions. Exceptions: in a few experiments where blinding was possible, the person performing the experiment was in fact blinded to the samples/conditions. These include: the service that performed the ChIP-qPCR being blinded to what each primer pair was measuring, and the core services conducting the Illumina sequencing for both genome editing and RNAseq analysis being blinded to the sample conditions in each case.

Reporting for specific materials, systems and methods

We require information from authors about some types of materials, experimental systems and methods used in many studies. Here, indicate whether each material, system or method listed is relevant to your study. If you are not sure if a list item applies to your research, read the appropriate section before selecting a response.

Materials & experimental systems

n/a	Involved in the study
[ ]	[x] Antibodies
[ ]	[x] Eukaryotic cell lines
[x]	[ ] Palaeontology and archaeology
[x]	[ ] Animals and other organisms
[x]	[ ] Clinical data
[x]	[ ] Dual use research of concern
[x]	[ ] Plants

Methods

n/a	Involved in the study
[x]	[ ] ChIP-seq
[ ]	[x] Flow cytometry
[x]	[ ] MRI-based neuroimaging

Antibodies

Antibodies used	Mouse Anti-phospho-Histone H2A.X Ser139 Antibody, clone JBW301, Sigma #05-636. Rabbit Anti-53BP1 Antibody, Novus #100-305. Goat anti-Mouse IgG H+L 568, Invitrogen #A-11031; Goat anti-Rabbit IgG H+L 488, Invitrogen #A-11034. DAPI: Thermo #62248. Rabbit anti-Ki67 (1:100, Abcam #ab16667), rat anti-NeuN (1:500, Abcam #ab279297), rabbit anti-TUBB3/Tuj1 (1:500, Sigma #T2200). Goat anti-rabbit 488 (Invitrogen #A11008), goat anti-rat 488 (LifeTech #A11006), goat anti-rabbit 647 (Invitrogen #A21245). MRE11: Abcam ab230381. gH2AX: MilliporeSigma 07-164. All listed in methods.
Validation	Antibodies were used at the dilutions recommended by the manufacturer, then increased only if determined necessary by empirical experiments in Nussenzweig Lab (for DSB staining antibodies) and Conklin Lab (for neuronal purity antibodies). Final validated concentrations for each antibody (primary and secondary) for our iPSC-derived neurons are listed in full in the Methods section. Exception: MRE11 and gH2AX antibodies for ChIP-qPCR (listed above) were validated for ChIP in-house by ActiveMotif, the service which performed the ChIP-qPCR for us.

Eukaryotic cell lines

Policy information about cell lines and Sex and Gender in Research

Cell line source(s)	WTC-NGN2 and WTC-NGN2-CRISPRi iPSC cell lines were both generated by Kampmann Lab, from parental WTC iPSCs. WTC iPSC line is male, and available from Coriell as GM25256.
Authentication	WTC-NGN2 and WTC-NGN2-CRISPRi cells were validated by genotyping PCRs confirming stable integration of NGN2 in the AAVS1 safe harbor locus and of CRISPRi in the CLYBL safe harbor locus. Karyotyping confirmed normal karyotype. Furthermore, NGN2 integration was routinely confirmed by successful differentiation into neurons upon addition of doxycycline.
Mycoplasma contamination	All cell lines were routinely tested and confirmed mycoplasma-negative while in culture, throughout these experiments.
Commonly misidentified lines (See ICLAC register)	Our experiments did not use any cell lines listed in the ICLAC Register of Misidentified Cell Lines.

Plants

Seed stocks	N/A
Novel plant genotypes	N/A
Authentication	N/A

Flow Cytometry

Plots

Confirm that:

- [x] The axis labels state the marker and fluorochrome used (e.g. CD4-FITC).
- [x] The axis scales are clearly visible. Include numbers along axes only for bottom left plot of group (a 'group' is an analysis of identical markers).
- [x] All plots are contour plots with outliers or pseudocolor plots.
- [x] A numerical value for number of cells or percentage (with statistics) is provided.

Methodology

Sample preparation	To dissociate neurons for flow cytometry, culture media was removed and then neurons were washed gently with PBS. Papain (reconstituted to 20U/mL in PBS, Worthington #LK003178) was added and incubated for 10 minutes at 37C: 500 or 125 µL papain per well of a 6- or 24-well plate, respectively. Papain was then quenched with DMEM (Corning #10-013-CV) with 10% FBS (eg Avantor #1500-500 or Cytiva #SH30071.03) at 3-5x the papain volume, and pipetted around the edges to lift and collect the sheet of neurons. Neurons were then pelleted, resuspended in 100-500 µL of PBS per sample, and triturated gently to singularize. These samples were passed through strainer-capped FACS tubes (eg Stellar Sci #FSC-9005)
Instrument	Attune NxT
Software	Flow cytometry data were collected on the Attune NxT and then analyzed in FlowJo.
Cell population abundance	Cells were not sorted/collected after flow cytometry. Abundance of each population (eg GFP+ vs GFP-) was at most 10,000 cells, as 10,000 events per sample was set as the target for collection.
Gating strategy	Initial FSC/SSC gating was used to exclude small cell debris / dead cells, then to exclude doublets/multiplets. Events/cells that passed these gates were passed through the GFP+/- gates shown in the figures. GFP+/- gating was set by first analyzing a negative control with no GFP signal (untransduced/untransfected neurons), and setting the gate just above the highest point of this population, such that the GFP+ fraction of the untreated neurons was ~0%.

☒ Tick this box to confirm that a figure exemplifying the gating strategy is provided in the Supplementary Information.
